# A Weak Temporal Association Between Multi-Week Geomagnetic Activity and Satellite-Derived Solar-Induced Chlorophyll Fluorescence Anomalies

**DOI:** 10.3390/biology15161415

**Published:** 2026-08-18

**Authors:** Andrey V. Kitashov

**Affiliations:** 1 Faculty of Biology, Shenzhen MSU-BIT University, Shenzhen 518100, China; kitashov@smbu.edu.cn; 2 Faculty of Biology, Lomonosov Moscow State University, Leninskie Gory, Moscow 119991, Russia

**Keywords:** geomagnetic variability, Dst, solar-induced chlorophyll fluorescence, SIF, OCO-2, vegetation, photosynthesis, temporal surrogates, cluster bootstrap, land cover, environmental association

## Abstract

Magnetic-field effects have been reported in controlled biological systems, but the relevance of weak geomagnetic variability to vegetation under natural conditions remains uncertain. We examined temporal associations between satellite-derived solar-induced chlorophyll fluorescence (SIF) and geomagnetic disturbance derived from the Disturbance Storm Time (Dst) index. We defined the sign-inverted Dst index, SII, as the sign-inverted daily mean Dst, so that stronger negative Dst excursions corresponded to larger positive SII values. The primary exposure was the trailing 28-day mean SII, excluding the day of the SIF observation. The primary analysis used Orbiting Carbon Observatory-2 (OCO-2) SIF at 771 nm with leave-one-year-out harmonic adjustment for the annual cycle and linear calendar-time trend. Sensitivity and robustness analyses examined a 21-day exposure, a more flexible cyclic-spline seasonal adjustment, spatial cluster bootstrap, and three temporal-surrogate null models. We also examined temperature and vegetation strata, land-cover and geographic controls, adjustment for an ECMWF Reanalysis version 5 (ERA5) surface solar radiation downwards (SSRD)-derived photosynthetically active radiation (PAR) energy proxy and vapour-pressure deficit, and comparisons with planetary Kp index, 10.7 cm solar radio flux index (F10.7), and supplementary SIF wavelengths. In the primary analysis, the 28-day trailing mean of SII was weakly negatively associated with SIF anomalies (Spearman ρ = −0.051; 95% cluster-bootstrap CI, −0.053 to −0.050), with an estimated linear change of −0.0381 residual-SIF units per 100 nT. Empirical *p*-values were 0.084 for year permutation, 0.011 for circular shift, and 0.001 for 30-day block permutation. The association remained negative at 21 days and in persistently vegetated cells, but its magnitude was substantially reduced with cyclic-spline adjustment (ρ = −0.013 in the pairwise-matched sample). Negative estimates were found in several vegetated land-cover classes, whereas estimates for the Barren land-cover class and Sahara geographic control were close to zero. The contribution of SII to explained variance remained below one percentage point across the examined temperature regimes. Overall, the results show a weak temporal association whose magnitude depends on analytical choices. Independent observational replication and controlled experiments are needed to determine whether it reflects a biological response to natural geomagnetic variability.

## Simple Summary

Natural geomagnetic disturbances produce small variations superimposed on the Earth’s main magnetic field, but whether these variations are related to plant activity under natural conditions remains unclear. We combined satellite measurements of solar-induced chlorophyll fluorescence, an indirect indicator of vegetation photosynthetic activity, with a global index of geomagnetic disturbance. Higher values of the 28-day trailing geomagnetic index were associated with slightly lower fluorescence anomalies. The association was small, became weaker when seasonal variation was removed using a more flexible method, and was not reproduced in the Barren land-cover class or in the Sahara geographic control. This observational pattern requires confirmation with independent satellite products and controlled experiments.

## 1. Introduction

Plant responses to magnetic fields have been studied under a wide range of experimental conditions. Reviews have reported changes in seed germination, growth and development, cell ultrastructure, mineral nutrition, gene expression, redox regulation, photosynthetic processes, and responses to environmental stress [1,2,3,4,5]. The evidence, however, remains heterogeneous. Studies differ substantially in field intensity, frequency, direction, waveform, exposure duration, biological material, environmental conditions, and measured outcomes. Many reported effects still lack independent replication, and no single molecular or biophysical mechanism has been shown to account for the full range of plant responses.

The broad term “magnetic-field effect” covers several physically and biologically distinct exposure regimes. These include strong static fields used for seed or plant treatment, weak static or alternating fields with intensities near the geomagnetic background, extremely low-frequency magnetic fields, substantial reduction or cancellation of the geomagnetic field, reversal of its direction, and comparatively small time-varying disturbances superimposed on the ambient geomagnetic field. Findings from one exposure regime cannot be assumed to apply to another because their amplitudes, frequencies, temporal structures, and possible interaction mechanisms differ [2,3,6,7,8].

Controlled experiments under near-null or hypomagnetic conditions form an important part of this literature. Studies in *Arabidopsis thaliana*, tomato, Lima bean, potato, and wild *Solanum* species have reported changes in growth and development, flowering and gene expression, root mineral nutrition, photosynthetic traits, redox responses, and metabolite profiles [9,10,11,12,13,14]. Studies have also examined developmental and transcriptional responses to reversal of the geomagnetic-field direction [15]. These findings indicate that substantial modification of the geomagnetic background can be associated with diverse plant responses under controlled conditions, but such exposures do not reproduce the much smaller disturbances superimposed on the ambient geomagnetic field.

Another line of experimental work has examined extremely low-frequency magnetic fields, including frequencies within or near the Schumann-resonance range. Reported responses include changes in plant water relations and growth, photosynthetic light reactions, redox metabolism, and light-induced electrical activity [16,17,18,19]. These studies are relevant because both natural and anthropogenic magnetic environments contain low-frequency components. Nevertheless, experimentally imposed oscillatory fields differ from geomagnetic storms in amplitude, spectral composition, waveform, duration, and temporal intermittency. Responses to a specific extremely low frequency (ELF) magnetic field therefore cannot be assumed to predict responses to multi-week geomagnetic variability associated with magnetic storms.

Natural geomagnetic variability represents another, comparatively less studied exposure regime. Minorsky reviewed historical and experimental evidence relating plant rhythms and physiological processes to geomagnetic pulsations, magnetic storms, and solar–geomagnetic periodicities [20]. Krylov and colleagues subsequently reproduced the waveform of a representative geomagnetic disturbance in the laboratory and examined biological responses to both the full disturbance and selected components [21]. More recent reviews have considered biological responses to magnetic storms, ELF magnetic fields, and hypomagnetic conditions across multiple taxa [7,8]. Together, these studies provide a basis for investigating natural geomagnetic variability, but plant-specific evidence remains sparse, methodologically diverse, and largely limited to controlled exposures or observations of individual organisms.

The unresolved question is not whether plants can respond to altered magnetic fields, but whether comparatively small, naturally occurring geomagnetic disturbances are detectably associated with plant function under field conditions. In particular, ecosystem-scale evidence linking nanotesla-range geomagnetic variability with photosynthesis-related observations remains very limited. Testing such an association requires observations spanning multiple years and broad spatial scales, together with careful consideration of seasonality, environmental covariation, temporal dependence, vegetation heterogeneity, and observational uncertainty.

The physical receptors and molecular pathways that might mediate weak-field responses still remain uncertain. The radical-pair mechanism seems to be the best-developed physical framework for explaining how weak magnetic fields might alter the spin dynamics and reaction yields of short-lived radical intermediates [22,23,24]. Cryptochromes are blue-light flavoproteins whose photochemistry makes them prominent candidates for radical-pair-based magnetic sensitivity [25,26]. Magnetic-intensity-dependent, cryptochrome-related responses have been reported in *Arabidopsis thaliana* [27], but the generality and mechanistic interpretation of cryptochrome-based magnetosensitivity remain debated [28,29]. Alternative or complementary hypotheses involving iron-containing structures, redox-active cofactors, distributed cellular sensing, or indirect effects on redox regulation have also been proposed [30,31,32]. None of these mechanisms can be inferred directly from ecosystem-scale remote-sensing data.

Geomagnetic activity is summarized using global or planetary indices. The disturbance storm time (Dst) index typically becomes negative during magnetic storms and is available through the OMNI solar wind and magnetic field data set [33]. In the present study, we define SII as the sign-inverted daily mean Dst, so that stronger negative Dst excursions correspond to larger positive SII values. SII therefore served as a temporal indicator of global geomagnetic disturbance. The planetary Kp index provides a related but differently constructed measure of geomagnetic activity [34], whereas the 10.7 cm solar radio flux serves as a comparator for broader solar variability [35].

To assess possible vegetation responses to such weak environmental variation, one would require observations with broad spatial and temporal coverage. Satellite-derived solar-induced chlorophyll fluorescence (SIF) provides one such measure. SIF originates from a small fraction of absorbed photosynthetically active radiation that is re-emitted by chlorophyll as fluorescence. Its magnitude reflects both absorbed radiation and the partitioning of excitation energy among photochemistry, non-photochemical dissipation, and fluorescence [36,37,38,39]. Satellite SIF has consequently become an important ecosystem-scale observable related to photosynthetic activity, vegetation function, and gross primary productivity [40,41,42].

The Orbiting Carbon Observatory-2 (OCO-2) provides repeated global SIF observations at far-red wavelengths with high spectral resolution [43,44,45]. The direct 771 nm retrieval was used as the sole primary SIF outcome. Restricting the primary analysis to one outcome reduced multiplicity and made the results easier to interpret, particularly because the expected association was small relative to the variability of individual satellite retrievals. The direct 757 nm retrieval and the provider-derived 740 nm variable were retained as supplementary sensitivity outcomes.

At the satellite scale, SIF integrates absorbed radiation, fluorescence yield, canopy structure, reabsorption, atmospheric transmission, solar and viewing geometry, phenology, vegetation density, water status, and plant functional type [38,39,42,44]. We therefore treated SIF as an ecosystem-scale feature and not a readout of a particular photosystem or molecular pathway.

Physiological and ecological conditions may influence how readily a weak association can be detected. Temperature influences carbon assimilation, photosynthetic electron transport, non-photochemical energy dissipation, respiration, redox balance, and the relationship between photochemical energy supply and metabolic demand [46,47,48]. These responses vary strongly among species, C3 and C4 pathways, plant functional types, climates, and acclimation histories [49,50,51]. We therefore used temperature strata to examine whether the association varied across environmental conditions.

The present study examines whether multi-week geomagnetic variability is temporally associated with satellite-derived SIF anomalies under natural conditions. We combined OCO-2 observations from September 2014 through December 2024 with Dst-derived SII exposures, ERA5 meteorological variables, Moderate Resolution Imaging Spectroradiometer (MODIS) leaf area index (LAI), and MODIS land-cover information on a global 0.5° × 0.5° grid [45,52,53,54,55]. The primary analysis used SIF at 771 nm, a fixed 28-day trailing mean of SII that excluded the day of the SIF observation, and leave-one-year-out harmonic adjustment for seasonality and linear calendar-time trend. A 21-day trailing mean and a leave-one-year-out cyclic-spline seasonal adjustment were used as sensitivity analyses.

We addressed four main questions. First, is the primary SII–SIF association stable under spatial cluster bootstrap and under temporal null models that preserve different components of the exposure time series? Second, does the association vary among temperature regimes, vegetation-density strata, land-cover classes, and geographic controls? Third, does the SII coefficient remain directionally consistent after simultaneous adjustment for photosynthetically active radiation and vapour-pressure deficit, and how much does it contribute to explained variance? Fourth, is the pattern reproduced by related geomagnetic and solar indices or by supplementary SIF wavelengths?

The study was exploratory and was intended to determine whether any observed temporal association was consistent enough to justify independent cross-sensor replication and controlled experiments using environmentally realistic magnetic perturbations.

## 2. Materials and Methods

### 2.1. Study Design and Analytical Hierarchy

We conducted an exploratory observational analysis of temporal associations between global geomagnetic activity and satellite-derived solar-induced chlorophyll fluorescence (SIF).

The primary specification used OCO-2 SIF at 771 nm, the 28-day trailing mean of SII, and leave-one-year-out harmonic adjustment for seasonality and linear calendar-time trends. The 28-day window was treated as an operational four-week exposure definition rather than as an empirically identified physiological timescale. A 21-day window and leave-one-year-out cyclic-spline seasonal adjustment were used as sensitivity analyses. The 1–90-day window scan was descriptive and was not used to choose the primary exposure window or an optimal lag.

Analyses by temperature, LAI, land cover, geographic and low-vegetation controls, environmental adjustment, alternative geomagnetic and solar indices, and supplementary SIF wavelengths were exploratory, descriptive, or diagnostic. For inference, we combined spatial cluster-bootstrap confidence intervals with three temporal-surrogate null models. The analytical hierarchy is summarized in Table S11. Observation eligibility did not depend on the sign or magnitude of the observed SII–SIF association.

### 2.2. Data Sources and Spatial–Temporal Harmonization

The analysis covered September 2014 through December 2024. Fluorescence observations were obtained from the OCO-2 Level 2 Lite retrospective SIF products, versions V11r and V11.2r [45]. Raw oco2_LtSIF_*.nc4 files were processed to select valid land observations and aggregate accepted soundings to daily cell means. Instrument performance, calibration, retrieval validity, and viewing-geometry dependence have been described previously [43,44]. Direct SIF retrievals at 757 and 771 nm were included, with the 771 nm retrieval used as the primary outcome.

Geomagnetic and solar indices were obtained from the NASA OMNI collection [33,56]. Dst was available at hourly resolution, Kp at three-hourly resolution, and F10.7 cm solar radio flux at daily resolution. Kp is a standard planetary index derived from mid-latitude geomagnetic observatories [34], whereas F10.7 is a widely used proxy for solar activity and solar extreme-ultraviolet variability [35].

ERA5 reanalysis provided hourly standard 2 m air temperature, standard 2 m dew-point temperature, and surface solar radiation downwards (SSRD) [52,53]. ERA5 variables were aggregated to daily values on the analytical grid. The PAR variable was an energy proxy calculated as 0.45 × SSRD/3600 for each hourly record and then averaged daily. It was expressed in W m^−2^ and was not interpreted as photon-flux PAR. Vapour-pressure deficit (VPD) was calculated from 2 m temperature and dew-point temperature using the Magnus formula and was expressed in kPa.

MODIS/Terra MOD15A2H Collection 6.1 eight-day composites supplied leaf area index (LAI) [54]. The MOD15A2H data were distributed on a regular geographic 0.5° grid whose cell centres were offset by 0.25° from those of the analytical grid. The dominant 2022 MCD12C1 Collection 6.1 IGBP land-cover classification was used for land-cover analyses [55].

All spatial datasets were referenced to a common 0.5° × 0.5° OCO-2/ERA5 grid with cell centres on integer and half-degree coordinates. MOD15A2H data were transferred from the offset 0.5° grid by equal-overlap remapping between the two regular 0.5° grids. Coordinate rounding and floor-based nearest-cell reassignment were not used. The MODIS quality flag was used as a diagnostic categorical variable and was not interpolated as a continuous variable.

For the primary analysis, MODIS data were linked to SIF observations using a central-only exact match that required the same analytical cell and the same eight-day composite period. No spatial or temporal fallback matching was used for the primary input; hierarchical matching was retained only as a sensitivity analysis. The central-only match covered more than 99% of otherwise eligible observations. Observations without a valid exact cell/composite match were excluded. We assigned dominant MCD12C1 land cover to each 0.5° analysis-grid cell.

For each model, the analytical sample included all observations meeting the applicable quality-control, exposure-availability, detrending, and complete-case requirements. Sample sizes varied slightly across specifications because of differences in variable availability and pairwise matching. The corresponding numbers of observations and spatial cells are reported with each estimate and summarized in Table S2.

### 2.3. SIF Outcomes and Quality Control

The primary outcome was OCO-2 SIF at 771 nm. SIF at 757 nm was analyzed as a supplementary direct spectral retrieval. The 740 nm SIF variable supplied in the source product was treated as a provider-derived estimate rather than as an independent instrumental retrieval [45]. Agreement among the 771, 757, and 740 nm results was therefore interpreted as internal spectral consistency.

The SIF input was restricted to land retrievals. Initial eligibility criteria required cloud fraction ≤ 60%, aerosol fraction ≤ 50%, LAI ≥ 0.01, and absolute latitude < 60°. The rule sif_771 nm ≥ 0.001 was used only as a sensitivity flag. No additional observations were removed as statistical outliers on the basis of SIF or SII magnitude. Non-finite SIF values and observations lacking the parameters required for a specific analysis were excluded from that analysis.

### 2.4. Geomagnetic and Solar Exposure Variables

Hourly Dst values from OMNI were converted to daily means [33]. The geomagnetic exposure index was defined operationally as

$$SII(t)=-{\bar{Dst}}_{daily}(t).$$

SII was treated as a global temporal indicator of geomagnetic disturbance; it does not quantify the local magnetic-field vector, local disturbance amplitude, or geographic differences in the field experienced by vegetation within individual analysis cells.

For a trailing window of *w* days, the daily SII series was shifted by one day and averaged over the preceding *w* days (the day of observation excluded from its own exposure window):

$${SII}_{w}(t)=\frac{1}{w}\sum_{k=1}^{w}SII(t-k).$$

The 28-day trailing mean was the primary exposure. The 21-day trailing mean served as the shorter-window sensitivity exposure.

Kp values in the source series were stored in integer units corresponding to 0.1 Kp and were multiplied by 0.1 before analysis. Daily Kp and F10.7 series were processed using the same one-day shift and trailing-mean rules as SII. Kp was treated as a related planetary geomagnetic index, not an independent replication of SII [34], whereas F10.7 served as a comparator for broader solar variability [35]. Whenever exposure availability differed, direct comparisons used matched observation sets.

### 2.5. Leave-One-Year-Out Seasonal Adjustment

We used two deliberately contrasting leave-one-year-out approaches. SII was not used as an explicit predictor or as a criterion for fitting either seasonal baseline. However, both models are time-based; consequently, they can inadvertently absorb SII-related signals that coincide with the modelled seasonal structure.

The primary harmonic model accounted for a continuous calendar-time trend and a fixed annual sine–cosine cycle. The resulting residuals represented deviations from this simple annual baseline and retained nonlinear phenology, interannual ecological variation, and other temporal variation not captured by the model.

The cyclic-spline model provided a more flexible empirical seasonal adjustment and was used as a sensitivity analysis. Contrary to the harmonic model, the spline treats the within-year pattern as a smooth function of calendar phase. The spline therefore could not distinguish nonlinear phenology, environmental covariation, sampling structure, or a possible SII-associated component with a similar temporal pattern: if such a component followed a temporal pattern that the spline could represent, it could be removed along with seasonal variation. The resulting residuals mainly retained variation not captured by this flexible annual baseline.

For each target year, observations from that year were excluded from fitting. The fitted model was then used to predict SIF for the held-out observations.

#### 2.5.1. Harmonic Model

The primary seasonal-adjustment model is

$$SIF(t)=\beta_{0}+\beta_{1}T(t)+\beta_{2}sin\left(\frac{2\pi \;DOY(t)}{365.25}\right)+\beta_{3}cos\left(\frac{2\pi \;DOY(t)}{365.25}\right)+\varepsilon (t),$$

where T(t) is centred continuous calendar time and DOY(t) is day of year. The SIF anomaly used in subsequent analyses was the observed value minus the leave-one-year-out prediction.

#### 2.5.2. Cyclic-Spline Sensitivity Model

The sensitivity model is

$$SIF(t)=\beta_{0}+\beta_{1}T(t)+f_{cyclic}\left[\phi (t)\right]+\varepsilon (t).$$

where ϕ(t)∈[0,1) represents annual phase and $f_{cyclic}$ is a cyclic B-spline basis with eight degrees of freedom. The model used the same leave-one-year-out procedure and the same minimum requirements for observations and training years as the harmonic model.

#### 2.5.3. Pairwise-Matched Comparison

The harmonic and cyclic-spline procedures produced slightly different sets of valid residuals. Their direct comparison therefore used a pairwise-matched sample restricted to observations with valid residuals under both methods. This reduced the possibility that differences between the two estimates simply reflected differences in observation availability.

### 2.6. Analytical Samples and Control Definitions

The analytical samples and controls described below were defined without reference to the sign or magnitude of the observed SII–SIF association. Their operational definitions and analytical roles are summarized in Table 1, and their spatial distributions are shown in Figure S1.

### 2.7. Temperature Stratification

Temperature regimes were assigned using the trailing 10-day mean of ERA5 2 m air temperature:

- Frozen: T < −1 °C;
- Cold: −1 °C ≤ T ≤ 7 °C;
- Cool: 7 °C < T ≤ 15 °C;
- Optimum: 15 °C < T ≤ 25 °C;
- Warm stress: 25 °C < T ≤ 30 °C;
- Extreme heat: T > 30 °C.

The cut points defined broad operational environmental strata. The labels were descriptive shorthand, and the boundaries were not derived from species-specific thermal-response curves. We used the temperature strata to examine ecological heterogeneity in the association.

### 2.8. LAI Stratification

Cell-level LAI distributions were calculated from the remapped MOD15A2H series [54]. In addition to the persistent-vegetation and strict low-LAI criteria defined above, cells were divided into quartiles according to their median LAI. Quartile boundaries were calculated from the cell-level LAI distribution and applied using the original unrounded cut points stored in the analysis data.

LAI-stratified analyses used the primary 28-day trailing SII exposure, harmonic residuals, spatial cluster-bootstrap confidence intervals, and the three temporal-surrogate models. The quartile analysis was exploratory and was used to examine ecological heterogeneity, not to infer a dose–response relationship.

### 2.9. Land-Cover Classification

The dominant 2022 MCD12C1 IGBP class was assigned to each 0.5° analysis-grid cell [55]. The native classes were grouped into Barren, Shrubland/Savanna, Savanna, Grassland, Cropland, Forest, and Other/unclassified categories. Cells in the Other/unclassified category were retained in the full sample but were not analyzed as a separate land-cover class for inference.

We estimated pooled 28-day associations by land-cover class without further temperature stratification. Harmonic and cyclic-spline estimates and the three temporal-surrogate *p*-values were reported by class. Because land cover also covaries with latitude, climate, moisture, phenology, and satellite sampling density, we interpreted differences among classes as descriptive ecological heterogeneity rather than as evidence of land-cover-specific biological effects.

### 2.10. Association and Effect-Size Estimates

Association strength was summarized by Spearman’s rank correlation between the detrended SIF residuals and the trailing SII exposure:

$$\rho ={cor}_{Spearman}\left({SIF}_{residual},{SII}_{w}\right).$$

Spearman’s rank correlation was used as the primary association measure because it does not assume normally distributed variables and is less sensitive to extreme values than Pearson correlation. Spatial and temporal dependence were addressed separately using spatial cluster bootstrap and temporal-surrogate inference.

Ordinary least-squares models were used to obtain descriptive linear slope estimates:

$${SIF}_{residual,i}=\alpha +\beta_{SII}{SII}_{w,i}+\varepsilon_{i}.$$

For the main fixed-window summaries, the unstandardized coefficient was expressed as the change in residual SIF associated with a 100 nT difference in trailing-mean SII:

$$\Delta {SIF}_{100\;nT}=100\;\beta_{SII}.$$

where standardized coefficients were reported in environmental-driver or variance-decomposition tables, the SII coefficient was expressed for a one-standard-deviation difference in SII and in units of the standard deviation of the SIF residual. Nominal *p*-values from Spearman correlations and OLS models were retained only as descriptive diagnostics and were not used for primary inference because the observations were spatially and temporally dependent.

**Figure 1:**
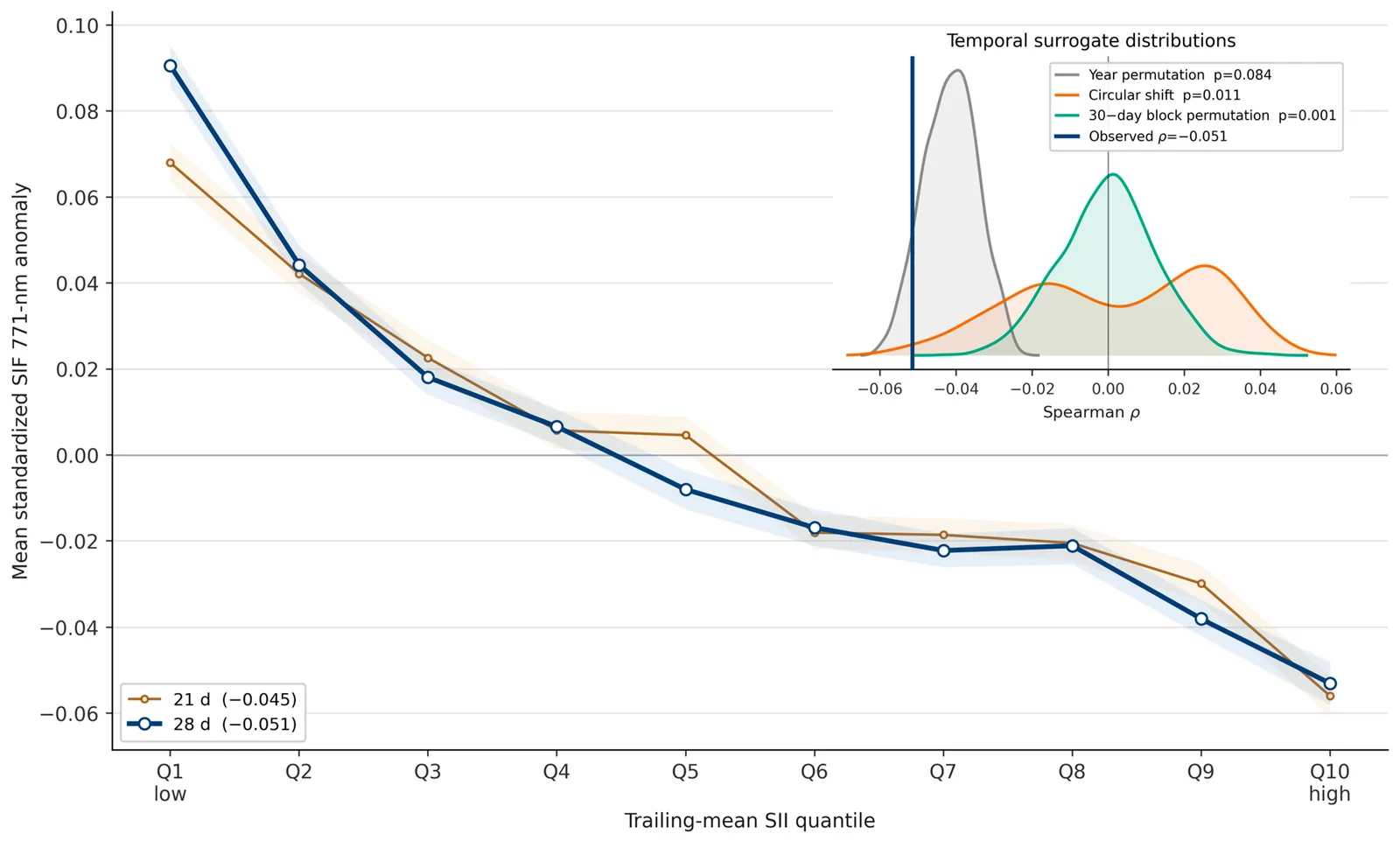
Primary association between the 28-day trailing mean of the Dst-derived geomagnetic index and OCO-2 SIF anomalies, with temporal-surrogate inference. Residual OCO-2 SIF at 771 nm after leave-one-year-out harmonic adjustment is summarized across deciles of the 28-day trailing mean of SII. The exposure window covers days t − 28 through t − 1, excluding the day of the SIF observation. Decile summaries are shown for visualization only; all reported estimates were calculated from the continuous exposure (Spearman ρ = −0.051; 95% spatial cluster-bootstrap CI, −0.053 to −0.050; OLS slope, −0.0381 residual-SIF units per 100 nT). In the main panel, shaded areas indicate 95% spatial cell-cluster bootstrap confidence intervals. The inset compares the observed Spearman correlation with null distributions generated from 1000 year permutations, 1000 leap-year-aware circular shifts, and 1000 permutations of 30-day blocks. Two-sided plus-one empirical *p*-values were 0.084, 0.011, and 0.001, respectively. The three null models preserve different aspects of temporal structure and are reported separately. Their maximum empirical *p*-value is shown only as a conservative summary and is not a formal combined *p*-value.

For visualization of the primary association in the main panel of Figure 1, the continuous 28-day trailing-mean SII exposure was divided into deciles (Q1–Q10), and residual SIF was summarized within each decile. This grouping made the pattern across the exposure range easier to display without imposing a parametric response curve. The deciles were used only for visualization; all Spearman correlations, slope estimates, bootstrap confidence intervals, and empirical *p*-values were calculated from the continuous exposure values.

### 2.11. Spatial Cluster-Bootstrap Confidence Intervals

Uncertainty in Spearman ρ was quantified using a spatial cluster bootstrap, with the 0.5° analysis cell rather than individual daily observations as the resampling unit [59]. For each realization, cells were sampled with replacement, and all temporal observations from each selected cell were retained before Spearman ρ was recalculated.

Each estimate was based on 1000 bootstrap realizations using a fixed stage-specific random seed. The 95% confidence interval was defined by the 2.5th and 97.5th percentiles of the bootstrap distribution. These intervals describe stability to spatial resampling of analysis cells; they do not test whether the global SII series is unusually aligned in time with the SIF residuals. Very small subsets of cells, including the strict low-LAI control, were therefore treated descriptively rather than interpreted using conventional sample-level *p*-values. Fixed-window estimates, linear slope summaries, and cluster-bootstrap confidence intervals are provided in Supplementary Data S1.

### 2.12. Temporal-Surrogate Inference

Temporal-surrogate tests generated alternative daily SII series under three structured null models and compared the observed Spearman statistic with the corresponding surrogate distributions [60]. After each transformation, the one-day shift and trailing-mean exposure were recalculated using exactly the same procedure as for the observed SII series.

#### 2.12.1. Year Permutation

Whole calendar years were randomly reassigned, with leap years and non-leap years permuted separately. This null preserved the temporal organization within each year while disrupting the alignment of individual geomagnetic years with the SIF observation record.

#### 2.12.2. Circular Shift

The complete daily SII series was circularly shifted by a leap-year-aware random offset of at least 30 days. This null preserved the sequence and autocorrelation of the SII series while disrupting its alignment with the observed SIF dates.

#### 2.12.3. Thirty-Day Block Permutation

The daily SII series was divided into 30-day blocks, and the blocks were randomly reordered. This null preserved short-range temporal organization within blocks while disrupting longer-range ordering.

Each null model used *B* = 1000 realizations. Two-sided empirical *p*-values were calculated using the plus-one convention:

$$p_{emp}=\frac{1+\sum_{b=1}^{B}I\left(\left|\rho_{b}|\geq |\rho_{obs}\right|\right)}{B+1}.$$

The minimum attainable empirical *p*-value was therefore 1/1001 ≈ 0.001. All three *p*-values were reported because the null models preserve different aspects of the temporal structure. For compact tabular summaries, we also reported the largest of the three empirical *p*-values:

$$p_{max}=max\left(p_{year},p_{shift},p_{block}\right).$$

This value was used only as a conservative single-number summary; it was not treated as a formal combined *p*-value or as an additional statistical test.

### 2.13. Descriptive Exposure-Window Analysis

We performed a descriptive scan of SIF at 771 nm across SII trailing windows from 1 to 90 days. Temporal alignment was fixed throughout: every exposure excluded the day of the SIF observation and used only the preceding days. For each window, we summarized the Spearman correlation, linear slope estimate, number of observations, and number of spatial cells for the harmonic residuals, cyclic-spline residuals, and pairwise-matched sample.

The 28-day primary window and the 21-day sensitivity window were defined separately from this descriptive scan. Results from the 1–90-day scan were not used to redefine either window, select the strongest correlation, or identify a minimum *p*-value. The scan was used only to show how the association varied with window length and was not interpreted as evidence for a unique biological response time. Results are shown in Figure S2 and Supplementary Data S3.

### 2.14. Environmental-Driver Adjustment

We examined the SII coefficient after simultaneous adjustment for PAR and VPD using

$${SIF}_{residual,i}=\alpha +\beta_{SII}{SII}_{w,i}+\beta_{PAR}{PAR}_{u,i}+\beta_{VPD}{VPD}_{v,i}+\varepsilon_{i}.$$

PAR and VPD were included as broad, spatially and temporally consistent covariates representing radiative energy supply and atmospheric evaporative demand, respectively. This analysis was not intended as an exhaustive environmental model; it tested whether the estimated SII coefficient depended strongly on the choice of PAR or VPD accumulation window.

Models were fitted separately by analytical scenario, temperature bin, and combination of SII, PAR, and VPD windows. SII windows ranged from 1 to 28 days. For each SII window, the PAR and VPD trailing windows varied independently from 1 to 28 days, giving 28 × 28 = 784 PAR–VPD combinations. Across these combinations, the distribution of β_SII_ was summarized by its median, interquartile range, fraction of negative estimates, and number of valid models.

This matrix was used to assess sensitivity to environmental-window choice rather than to select the window combination that produced the strongest SII association.

### 2.15. Shapley/LMG Variance Decomposition

We examined the relative contributions of SII, PAR, and VPD using a common 28-day trailing window:

$${SIF}_{residual,i}=\alpha +\beta_{1}{SII}_{28,i}+\beta_{2}{PAR}_{28,i}+\beta_{3}{VPD}_{28,i}+\varepsilon_{i}.$$

The full-model R^2^ was decomposed using the exact Shapley or Lindeman–Merenda–Gold (LMG) procedure [61]. With three predictors, all six possible predictor-entry orders were evaluated, and each predictor’s incremental contribution was averaged across those orders. Contributions were expressed as percentage points of R^2^ and summed to the full-model R^2^, apart from numerical rounding.

The decomposition describes variance allocation within the specified model; the predictors were temporally structured, mutually correlated, and potentially related to unmeasured environmental processes.

### 2.16. Kp, F10.7, and SAA Robustness Analyses

The primary 28-day SII association was compared with corresponding 28-day trailing means of Kp and F10.7 and with the SII association within the SAA geographic diagnostic. The Kp and F10.7 analyses used the same detrended SIF outcome, the same one-day exclusion of the observation day before calculating trailing means, and the same matched-sample rules. When exposure availability differed, the SII reference estimate was recalculated using the same set of observations.

These comparisons were treated as sensitivity analyses rather than as independent replications. Kp and SII are related measures of planetary geomagnetic activity, whereas F10.7 served as a comparator for solar radio emission and broader solar variability [34,35].

### 2.17. Supplementary SIF Wavelength Analyses

SIF at 757 nm and the provider-derived 740 nm estimate were analyzed as supplementary outcomes using the 21- and 28-day SII trailing means, harmonic and cyclic-spline residuals, pairwise-matched comparisons, Spearman correlations, linear slope estimates, and the temporal-surrogate framework [45].

Because the 740 nm variable was derived by the data provider from the direct SIF retrievals, it did not provide an independent wavelength or sensor replication. The 757 and 771 nm retrievals also came from the same instrument and shared atmospheric, spatial, temporal, and sampling structure [43,44].

### 2.18. Missing Data, Software, and Reproducibility

No missing SIF outcomes or seasonal residuals were imputed. Analyses used complete cases for the variables required by each model, so sample size could vary with the availability of covariates or alternative exposure variables. When seasonal-adjustment methods or exposure indices were compared directly, both estimates were restricted to the same pairwise-matched observations. These matching steps were based on data availability, not on the strength or direction of the observed SII–SIF association.

All analyses were performed in Python 3.11.15. Random procedures used fixed seeds, and analysis definitions and parameter values were kept in version-controlled configuration and source files. The code, parameter settings, and scripts used to generate the reported tables and figures are publicly available through the project repository 10.5281/zenodo.21768818.

## 3. Results

### 3.1. Analytical Coverage

After quality control and harmonization, the primary analysis using harmonic leave-one-year-out residuals and the 28-day trailing SII exposure included 1,949,342 daily observations from 24,304 spatial cells at 0.5° × 0.5° resolution. The cyclic-spline pairwise-matched sample included 1,947,618 observations from 24,299 cells. The spatial distribution of the full sample, persistently vegetated cells, the Barren land-cover class, and the geographic control regions is shown in Figure S1. Data coverage, quality-control criteria, and definitions of the analytical samples are summarized in Tables S1 and S2.

### 3.2. A Weak Negative Association Between the 28-Day Trailing Geomagnetic Index and SIF Anomalies

Residual SIF decreased slightly across increasing deciles of the 28-day trailing-mean SII exposure (Main panel of Figure 1). In the full harmonic sample, the continuous association was negative (Spearman ρ = −0.051; 95% cluster-bootstrap CI, −0.053 to −0.050), with an estimated linear slope of −0.0381 residual-SIF units per 100 nT increase in trailing-mean SII (Table 2). The large sample size notwithstanding, the association itself was small.

Temporal-surrogate outcomes varied with the null model’s retention of time-series features (Inset of Figure 1). For the primary 28-day harmonic analysis, the empirical *p*-values were 0.084 for year permutation, 0.011 for circular shift, and 0.001 for 30-day block permutation. The maximum empirical *p*-value across the three null models was therefore 0.084. Thus, the primary association was not uniformly supported across the temporal null models: the observed correlation was more extreme than the circular-shift and block-permutation distributions but was indistinguishable from the year-permutation null (Table S4; Figure S3; Supplementary Data S2).

The association remained negative for the 21-day sensitivity window (Spearman ρ = −0.045; 95% cluster-bootstrap CI, −0.047 to −0.044), with an estimated linear slope of −0.0315 residual-SIF units per 100 nT and a maximum empirical *p*-value of 0.128 (Table 2). The descriptive 1–90-day scan showed a broad multi-week region of negative estimates rather than a narrow isolated optimum and did not identify a uniquely preferred exposure window (Figure S2; Supplementary Data S3).

More flexible seasonal adjustment substantially attenuated the association. In the pairwise-matched cyclic-spline analysis at 28 days, the correlation remained negative (Spearman ρ = −0.013; 95% cluster-bootstrap CI, −0.014 to −0.012), with an estimated linear slope of −0.0091 residual-SIF units per 100 nT and a maximum empirical *p*-value of 0.127 (Table 2). The direction remained negative, but the magnitude shrank considerably relative to the harmonic adjustment.

Within the persistently vegetated sample, the primary harmonic association was also negative (Spearman ρ = −0.028; 95% cluster-bootstrap CI, −0.030 to −0.025), with an estimated linear slope of −0.0187 residual-SIF units per 100 nT and a maximum empirical *p*-value of 0.036 (Table 2).

Kp and F10.7 yielded weaker pooled harmonic correlations at 28 days (Spearman ρ = −0.033 and ρ = −0.012, respectively; Figure S5 and Table S5). For the supplementary SIF outcomes, harmonic estimates remained negative at 740 and 757 nm, whereas cyclic-spline estimates were near zero at 740 nm and weakly positive at 757 nm (Table S10). The provider-derived 740 nm variable was not an independent spectral measurement.

### 3.3. Temperature and Geographic Heterogeneity

**Figure 2:**
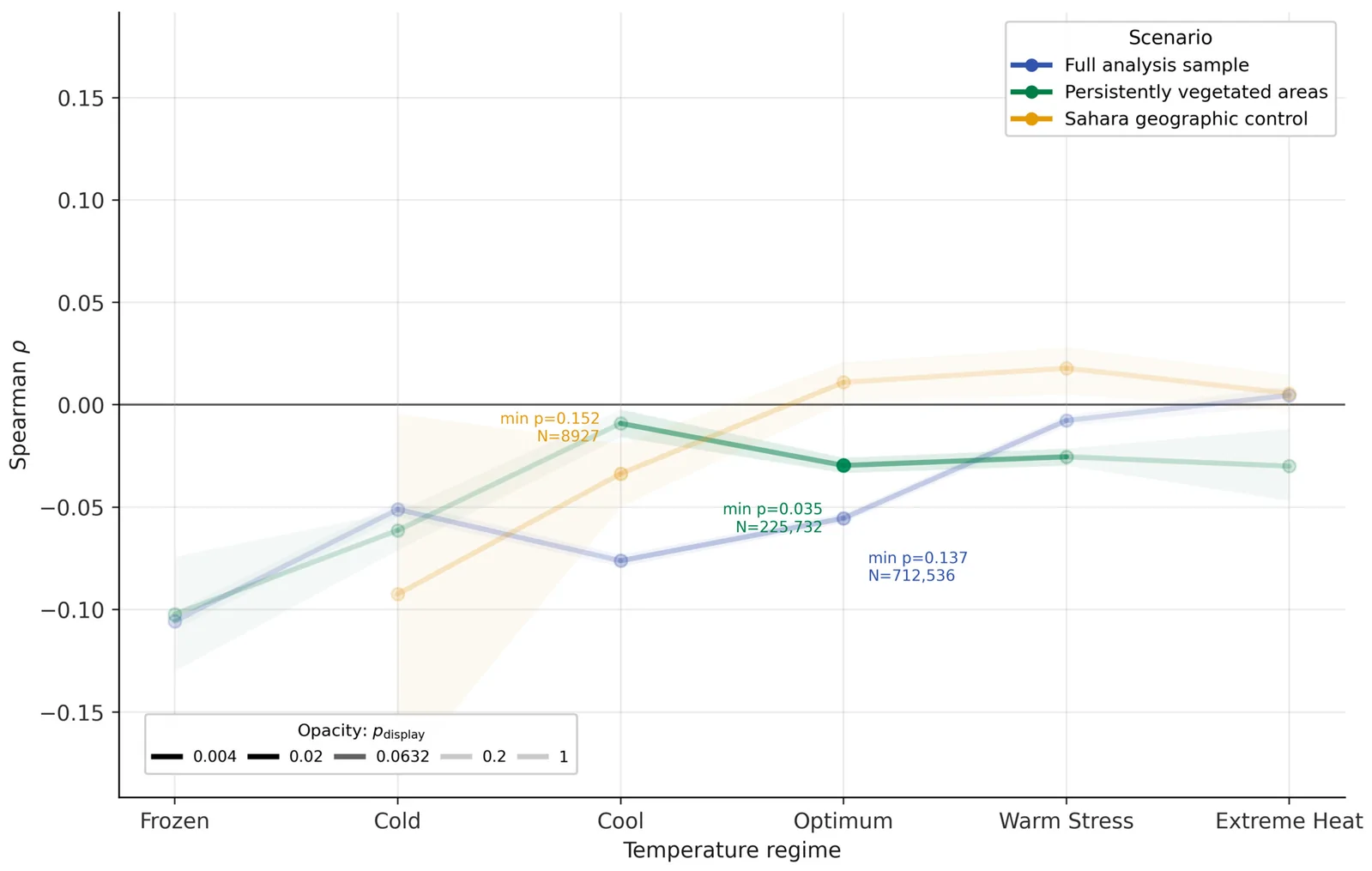
Temperature-stratified associations between the 28-day trailing mean of SII and OCO-2 SIF anomalies in the defined analytical samples. Spearman correlations were calculated from leave-one-year-out harmonic residuals within operational temperature regimes defined by the trailing 10-day mean of ERA5 2 m air temperature: Frozen (T < −1 °C), Cold (−1 °C ≤ T ≤ 7 °C), Cool (7 °C < T ≤ 15 °C), Optimum (15 °C < T ≤ 25 °C), Warm stress (25 °C < T ≤ 30 °C), and Extreme heat (T > 30 °C). Symbols and connecting lines identify the analytical samples shown in the legend, and uncertainty intervals are 95% spatial cluster-bootstrap confidence intervals obtained by resampling 0.5° spatial cells. In the pooled sample, Spearman ρ was −0.106, −0.051, −0.076, −0.055, −0.008, and +0.005 across the six regimes, respectively.

The SII–SIF association varied across temperature regimes and analytical samples (Figure 2). In the pooled 28-day harmonic analysis, Spearman ρ was −0.106 for Frozen, −0.051 for Cold, −0.076 for Cool, −0.055 for Optimum, −0.008 for Warm stress, and 0.005 for Extreme heat. Negative associations were more apparent in Frozen, Cold, Cool, and Optimum temperature observations and approached zero under Warm stress and Extreme heat.

Uncertainty increased in temperature bins represented by fewer spatial cells, especially at the climatic extremes. Full estimates, cluster-bootstrap confidence intervals, observation counts, and cell counts are reported in Table S3; the corresponding temporal-surrogate results are provided in Supplementary Data S2.

The Sahara geographic control remained close to zero across most temperature regimes and showed wider intervals than the full sample (Figure 2). In the pooled 28-day harmonic analysis, the Sahara association was small and positive (Spearman ρ = 0.012; 95% cluster-bootstrap CI, 0.006 to 0.018), with an estimated linear slope of 0.0019 residual-SIF units per 100 nT and a maximum empirical *p*-value of 0.842 (Table 2). This small estimate was not interpreted as evidence of a substantial ecological association.

The broader set of biological and geographic controls is shown in Figure S4. The strict low-LAI control contained very few spatial cells and was therefore treated descriptively. The South Atlantic Anomaly geographic diagnostic showed only a small pooled harmonic association (Spearman ρ = −0.014) and did not suggest that the primary result was dominated by a regional particle-related satellite artefact, although other instrumental or sampling effects remain possible (Tables S2 and S5).

LAI-based stratification provided an exploratory view of ecological heterogeneity. The analytical definitions are summarized in Table S9, while the quartile estimates, confidence intervals, sample sizes, and sample composition are shown in Figure S8 and Supplementary Data S6.

### 3.4. Adjustment for PAR and VPD and Decomposition of Explained Variance

The SII coefficient remained predominantly negative after simultaneous adjustment for PAR and VPD across a broad range of environmental window combinations (Figure S6; Table S6; Supplementary Data S4). For each SII window, the coefficient was evaluated across 784 combinations of PAR and VPD windows. Its predominantly negative sign across these combinations indicated that the association was not tied to a single choice of environmental window.

**Figure 3:**
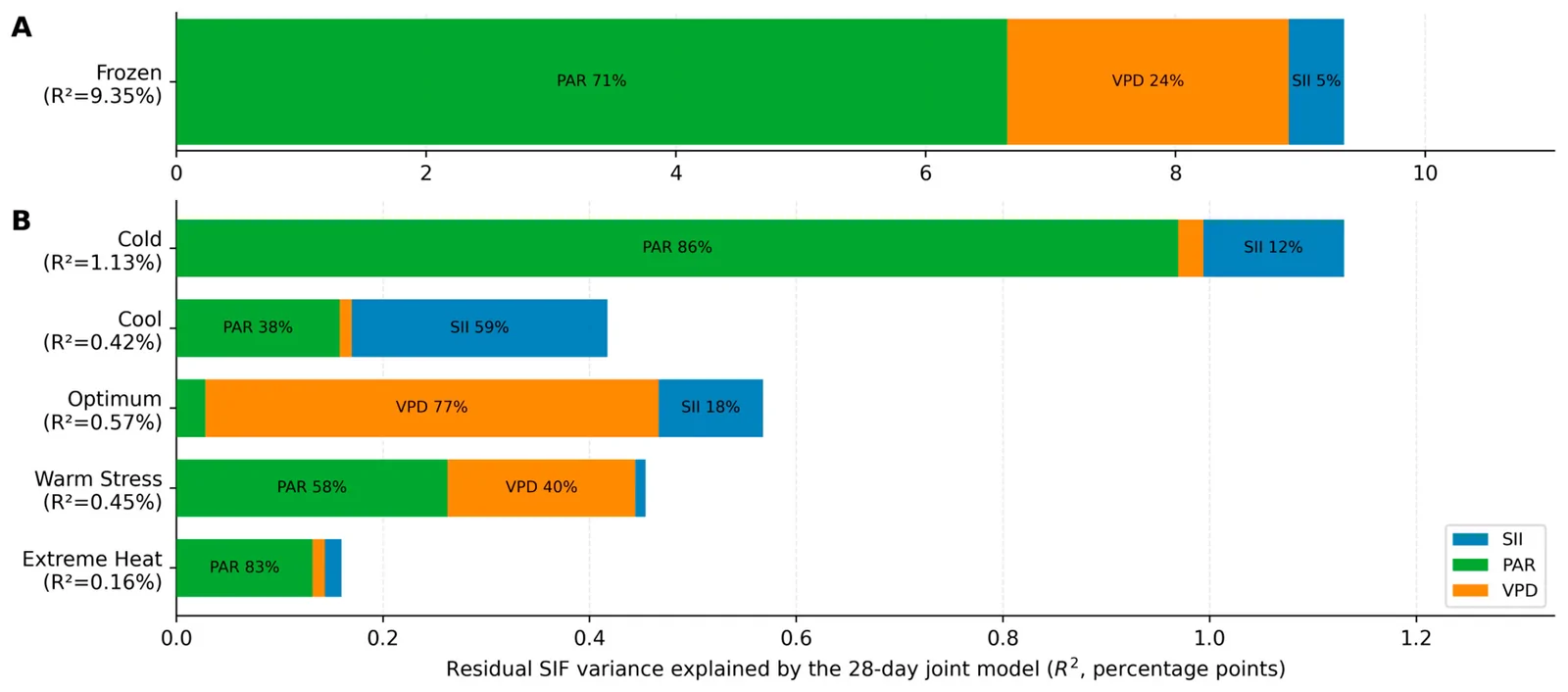
Shapley/LMG decomposition of explained residual-SIF variance for SII, PAR, and VPD at a common 28-day trailing window. Contributions are expressed as absolute R^2^ percentage points and sum to the full-model R^2^, apart from rounding. (**A**) Frozen regime, shown separately because of its substantially larger total model R^2^. (**B**) Cold, Cool, Optimum, Warm Stress, and Extreme Heat regimes. Percentages within the bars indicate the relative share of each predictor in the corresponding full-model R^2^. The absolute SII contribution remained below one percentage point in every temperature regime.

The Shapley/LMG decomposition assigned only a small absolute share of explained variance to SII, although its relative share varied substantially among temperature regimes (Figure 3; Table S7).

In the Frozen regime, the full three-predictor model explained 9.3488% of residual-SIF variance. PAR contributed 6.6512 percentage points, VPD 2.2553 percentage points, and SII 0.4423 percentage points. Thus, SII accounted for only a small fraction of the relatively large total model R^2^ under Frozen conditions.

In the Cool regime, the full model explained 0.4174% of the variance. The SII contribution was 0.2476 percentage points, compared with 0.1580 for PAR and 0.0118 for VPD. Although SII represented a relatively large share of this low total R^2^, its absolute contribution remained small.

In the Optimum regime, the full-model R^2^ was 0.5678%. VPD received the largest share (0.4390 percentage points), compared with 0.1008 percentage points for SII and 0.0279 for PAR. The SII contribution was also small in the Cold (0.1363 percentage points), Warm stress (0.0098 percentage points), and Extreme heat (0.0159 percentage points) regimes.

A relatively large Shapley share was not taken for a large absolute contribution to explained variance. Across most regimes, the total variance explained by the three predictors was low, and the absolute contribution assigned to SII remained below one percentage point. Complete temperature-stratified decompositions are provided in Table S7, Figure S7, and Supplementary Data S5.

### 3.5. Land-Cover Dependence of the Association

**Figure 4:**
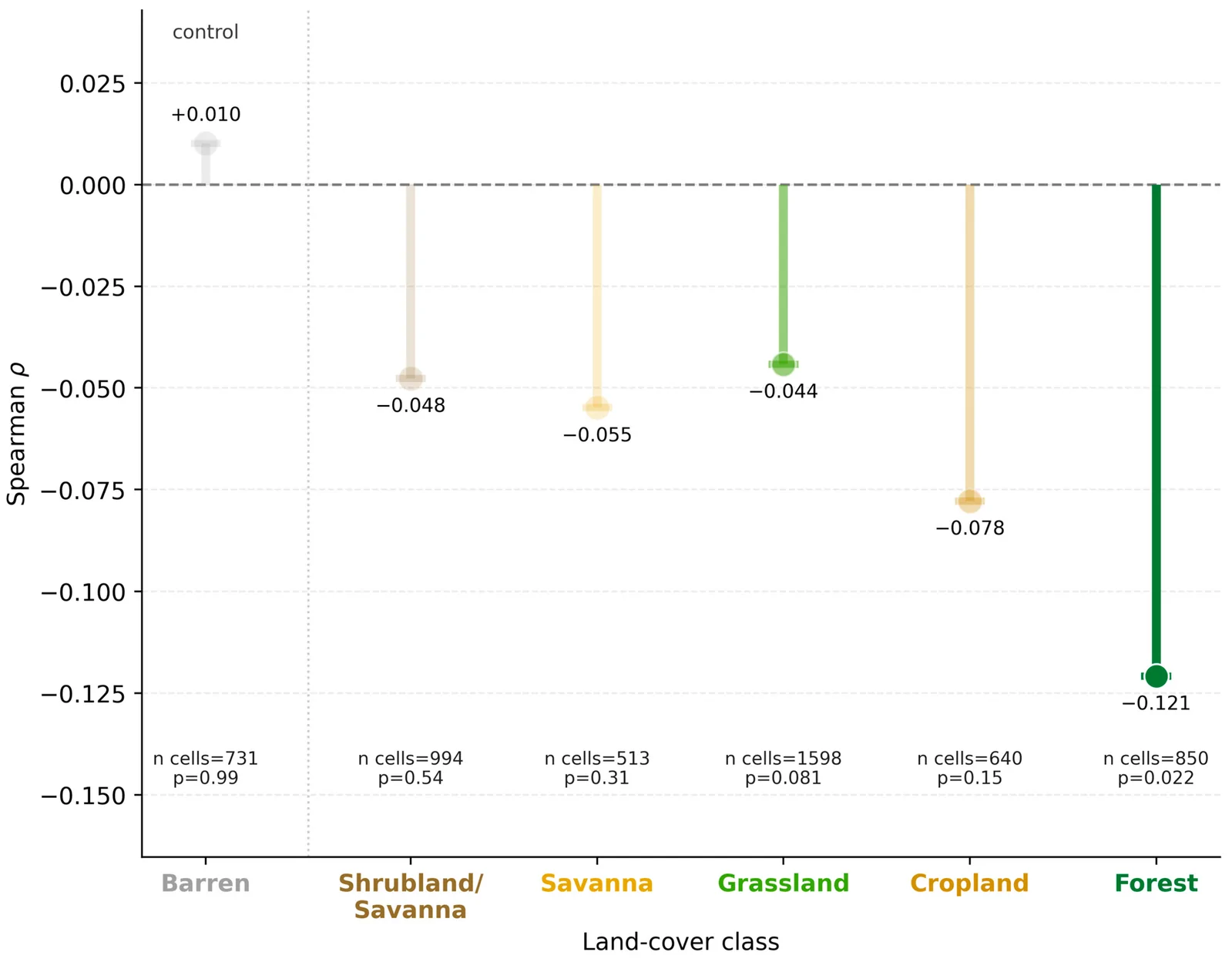
Association between the 28-day trailing mean of SII and OCO-2 SIF anomalies across dominant MCD12C1 land-cover classes. Spearman correlations were calculated from pooled leave-one-year-out harmonic residuals for the dominant 2022 MCD12C1 IGBP class assigned to each 0.5° analysis cell. Spearman ρ was −0.121 for Forest, −0.078 for Cropland, −0.055 for Savanna, −0.048 for Shrubland/Savanna, −0.044 for Grassland, and +0.010 for Barren. Vertical stems connect each estimate to zero and are not confidence intervals. The horizontal dashed line indicates ρ = 0. Opacity reflects the conservative temporal-surrogate p-value; the number of spatial cells and the corresponding *p*-value are shown below each class.

The pooled 28-day harmonic association differed among dominant land-cover classes (Figure 4). Spearman ρ was −0.121 for Forest, −0.078 for Cropland, −0.055 for Savanna, −0.048 for Shrubland/Savanna, −0.044 for Grassland, and 0.010 for the Barren land-cover class. Thus, several vegetated classes showed negative associations of differing magnitude, whereas the MCD12C1 Barren class remained close to zero.

Pooled estimates and sample sizes for each class are reported in Table S8; fixed-window land-cover estimates and the corresponding temporal-surrogate results are provided in Supplementary Data S6. Conventional parametric *p*-values were retained only as descriptive diagnostics and were not used for primary inference.

Land-cover classes also differed in temperature, moisture regime, LAI, phenology, latitude, and satellite sampling density. Their temperature and LAI distributions are shown in Figure S8 and Supplementary Data S6.

## 4. Discussion

### 4.1. Principal Findings

This study identified a weak negative temporal association between multi-week geomagnetic activity, represented by the sign-inverted Dst index (SII), and satellite-derived SIF anomalies. In the primary harmonic analysis, the 28-day trailing mean of SII was weakly negatively associated with SIF anomalies (Spearman ρ = −0.051), with an estimated linear slope of −0.0381 residual-SIF units per 100 nT. The association remained negative for the 21-day sensitivity window and within the persistently vegetated sample, but its magnitude was substantially reduced under cyclic-spline seasonal adjustment. Temporal-surrogate support also depended on the null model, with the primary association remaining compatible with the year-permutation null.

The key finding is not a robust geomagnetic influence, but a fragile negative association that is sensitive to analytical choices. Negative estimates were observed in the full and persistently vegetated samples and in several vegetated land-cover classes, whereas estimates for the Sahara geographic control and the Barren land-cover class were close to zero. Across temperature strata, the association was more negative in several lower and intermediate temperature regimes and weakened under warmer conditions, although the pattern was neither monotonic nor consistent with a simple temperature-response relationship.

The absolute explanatory contribution assigned to SII was small. In the Shapley/LMG decomposition, the contribution of SII remained below one percentage point of R^2^ in every temperature regime examined, and the full SII–PAR–VPD model generally explained only a small proportion of residual-SIF variance outside frozen conditions. These results are consistent with SII being, at most, a minor correlate within much larger radiative, hydrological, seasonal, structural, and physiological variability.

### 4.2. Robustness and Sensitivity to Analytical Assumptions

Several sensitivity analyses showed that the negative estimate was not confined to a single analytical choice. The spatial cluster-bootstrap intervals were narrow in the full and persistently vegetated samples, the direction remained negative at both 21 and 28 days, and the descriptive 1–90-day scan showed a broad region of negative multi-week estimates rather than an isolated optimum. The scan did not select the primary window, and the one-day offset was fixed *a priori*, not optimized. Observations were likewise not excluded on the basis of SIF or SII magnitude. Similar directions were seen for the supplementary SIF wavelengths, although the provider-derived 740 nm variable does not constitute independent spectral replication.

The temporal-surrogate results were less uniform. The primary harmonic association was more extreme than the circular-shift and 30-day block-permutation null distributions, but it was not clearly separated from the year-permutation distribution. The three null models therefore provided different levels of support because they preserved different components of temporal structure: year permutation retained within-year organization, circular shifting preserved the sequence and autocorrelation structure of the SII series, and block permutation preserved shorter-range dependence. The observed correlation fell in the tails of the circular-shift and block-permutation distributions but remained compatible with the year-permutation null.

The narrow spatial bootstrap interval and the less decisive temporal-surrogate inference address different sources of uncertainty. The cluster bootstrap evaluates how stable the estimate is to resampling spatial cells. It does not test whether temporally structured variation shared across many cells could produce an apparent alignment between SII and SIF. The surrogate tests address the temporal alignment of the exposure series with the observations. Statistical evidence should therefore not be summarized by the bootstrap interval alone.

Seasonal adjustment had a substantial effect on the estimated magnitude. In the same pairwise-matched observations, the 28-day Spearman correlation decreased from approximately −0.051 with harmonic adjustment to −0.013 with the cyclic spline, but remained negative. As the comparison was pairwise-matched, this attenuation cannot be attributed to differential sample availability between the two methods. The harmonic model may leave nonlinear phenological or environmental structure in the residuals, whereas the more flexible spline may absorb both such structure and any SII-associated variation with a similar temporal pattern. The observational data cannot distinguish between these possibilities. The effect size is demonstrably model-dependent, although its negative direction appears more robust.

The 28-day window should not be mistaken for a biologically validated timescale. Trailing means at neighbouring window lengths are strongly overlapping and autocorrelated and therefore do not represent independent experiments. The descriptive scan suggests a broad multi-week pattern, but it does not identify a unique optimum, response delay, or lifetime of an underlying magnetic interaction.

### 4.3. Environmental Adjustment and Residual Confounding

The SII coefficient remained predominantly negative after simultaneous adjustment for PAR and VPD across a broad range of environmental trailing-window combinations. This indicates that the estimated association was not restricted to a single choice of PAR or VPD window. The Shapley/LMG analysis likewise quantified the share of explained variance assigned to SII within the specified three-predictor model.

These analyses did not establish an effect independent from environmental forcing. SIF, PAR, VPD, temperature, cloud properties, atmospheric composition, phenology, soil moisture, canopy structure, solar geometry, and satellite sampling are components of an interconnected environmental system. A regression model containing PAR and VPD cannot represent every relevant pathway, interaction, or unmeasured covariate. SII, Kp, F10.7, atmospheric conditions, and seasonal vegetation dynamics may also share temporal structure without any direct magnetic influence on plants.

The comparison with F10.7 provides a check on whether a broader indicator of solar variability reproduces the SII pattern. The absence of an identical F10.7 pattern does not support the simple interpretation that any temporally structured solar index would yield the same association, but it does not exclude solar–atmospheric pathways that are incompletely represented by F10.7. Similarly, the Kp comparison shows whether a related planetary geomagnetic index exhibits a comparable pattern. Because SII and Kp are related measures of the same geophysical system, however, agreement between them cannot be regarded as independent replication [34,35].

### 4.4. Temperature, LAI, and Land-Cover Heterogeneity

The negative association was more apparent in lower and intermediate temperature regimes and weakened under warmer conditions. One possible physiological context for this pattern is that low temperature can increase the imbalance between photochemical energy input and the more temperature-sensitive biochemical use of reducing power. However, photosynthetic temperature responses vary substantially among species, functional types, climates, and acclimation histories [46,49,50,51].

The association spanned several contiguous temperature bands, suggesting gradual heterogeneity rather than a discrete threshold response. This suggests gradual heterogeneity across temperature conditions rather than a sharp threshold, but the observational analysis does not identify the physiological process responsible for that heterogeneity.

Leaf-area-index quartiles were used to explore whether canopy density modified the observed association (definitions in Table S9; estimates, intervals, and sample details in Figure S8 and Supplementary Data S6). These strata were not interpreted as a monotonic dose–response relationship. LAI reflects canopy quantity and persistence but does not uniquely characterize photosynthetic state, species composition, canopy geometry, productivity, or stress.

Land-cover results provided an additional ecological contrast. Forest, cropland, and grassland classes showed negative estimates of differing magnitude, whereas the Barren land-cover class remained close to zero. This contrast is compatible with an association involving vegetation-related processes, but it does not establish biological specificity. Land-cover classes also differ in latitude, climate, phenology, canopy structure, management, water availability, surface reflectance, and OCO-2 sampling. These correlated differences could likewise contribute to the class-specific estimates.

### 4.5. Interpretation of Satellite SIF

SIF is an integrated canopy-scale radiometric signal rather than a direct measurement of a specific photosystem, redox intermediate, or biochemical flux. It depends on absorbed radiation, fluorescence yield, canopy structure, reabsorption, atmospheric transmission, viewing geometry, phenology, and the partitioning of excitation energy among photochemistry, heat dissipation, and fluorescence [38,39,42,44].

Accordingly, the observed negative SII–SIF association does not by itself identify a specific physiological mechanism such as photosystem I inhibition, increased reactive oxygen species production, altered electron transport, or reduced carbon fixation. A biological response expressed through fluorescence could arise through several regulatory and structural pathways. At the same time, because SIF is a remotely sensed radiometric quantity, retrieval and sampling effects remain alternative contributors that cannot be fully excluded from the present observational analysis.

The supplementary SIF-wavelength analyses provide additional internal evidence for the observed pattern, although they do not constitute independent replication. The 757 and 771 nm retrievals originate from the same satellite platform and share observation times, atmospheric conditions, viewing geometry, and much of their processing, while the 740 nm estimate is derived by the data provider from the direct retrievals. The degree of agreement also depended on the seasonal-adjustment method. Independent measurements from another instrument, retrieval framework, or ground-based system would be needed to test the generality of the association beyond the present OCO-2 dataset.

The estimated linear slope cannot be translated directly into a change in gross primary production, biomass accumulation, or crop yield. The reported change in residual SIF per 100 nT quantifies the magnitude of the observed association within this dataset, while its ecological significance remains to be established.

### 4.6. Mechanistic Hypotheses

The observed association helps define conditions under which mechanistic experiments could be informative.

The radical-pair mechanism provides a general physical framework in which weak magnetic fields can alter spin-dependent reaction yields [23,24]. Plant cryptochromes are frequently discussed as candidate magnetic-field-sensitive flavoproteins, and cryptochrome-dependent responses to altered magnetic intensity have been reported under controlled conditions [27,29]. Other redox-active cofactors, including components of photosynthetic electron-transfer chains, have also been proposed as possible interfaces between magnetic-field perturbations and cellular regulation [32].

Redox-active quinones and reactive oxygen species can influence chlorophyll fluorescence and photosynthetic electron transport [62,63]. Such interactions offer a plausible, though speculative, conduit from weak magnetic perturbations to altered fluorescence. However, neither quinone redox state nor ROS production were measured in the present study. They therefore represent experimentally testable mechanistic candidates rather than mechanisms demonstrated by the satellite observations.

The present data do not identify which of these molecular pathways contributes to the observed SII–SIF association. No cryptochrome activity, radical-pair yield, iron–sulfur redox state, reactive oxygen species concentration, photosystem activity, or electron-transfer rate was measured directly. The satellite observations therefore cannot discriminate among cryptochrome-mediated signalling, other spin-dependent chemistry, redox-mediated regulation, or indirect physiological responses, nor can they by themselves exclude environmental or retrieval-related contributions.

The statistical multi-week exposure does not imply persistent spin-dependent chemistry; rather, it may reflect integrated effects of repeated short-lived perturbations. A biologically mediated multi-week association could instead arise from repeated short-lived magnetic perturbations whose effects are integrated through slower physiological processes, such as redox regulation, acclimation, antioxidant responses, canopy development, or changes in energy allocation. This provides a testable framework for controlled experiments rather than a claim that the 28-day window represents the lifetime of an underlying molecular process.

### 4.7. Geomagnetic Exposure Limitations

SII was defined from the sign-inverted daily Dst index. Dst is a global measure dominated by large-scale magnetospheric current systems; it does not represent the magnetic-field perturbation experienced at the location of an individual plant. Local geomagnetic variation depends on latitude, longitude, field component, ionospheric and magnetospheric currents, local time, conductivity structure, and the regional geomagnetic baseline.

Consequently, the estimated slope per 100 nT of SII should be interpreted as the change in residual SIF associated with a 100 nT difference in the global Dst-derived index, not as the response to a spatially uniform 100 nT field manipulation. The present study therefore characterizes temporal association with a global indicator of geomagnetic disturbance. It does not identify which field component, frequency range, direction, or local disturbance amplitude is most relevant to the observed association.

The Kp analysis provides a complementary test using a differently constructed planetary geomagnetic index, but both Kp and Dst remain large-scale summaries of geomagnetic activity. Future exposure models could more directly characterize the magnetic conditions experienced by vegetation by combining regional observatory records, gridded geomagnetic-field reconstructions, local disturbance amplitudes, and, where available, frequency-resolved field variations.

### 4.8. Instrumental and Geographic Controls

The Sahara geographic control and the Barren land-cover class did not show the negative association observed in several vegetated samples. The SAA diagnostic likewise provided no indication that the primary association was dominated by enhanced particle exposure in that geographic region. These contrasts argue against several simple geographic or instrument-related explanations of the observed pattern, although none constitute a definitive negative control.

The Sahara region has heterogeneous surface properties, substantial atmospheric dust, and limited but nonzero vegetation, and its OCO-2 sampling differs from that of many vegetated regions. The SAA mask was a rectangular geographic approximation and therefore could not capture the full spatial and temporal structure of particle exposure. Moreover, particle-related interference represents only one potential source of satellite bias. Cloud filtering, aerosols, solar and viewing geometry, surface reflectance, orbital sampling, and retrieval-quality variation could also introduce spatially or temporally structured artefacts outside the SAA [43,44,57,58].

### 4.9. Strengths and Limitations

A principal strength of the study is its explicit focus on weak natural geomagnetic variability rather than on experimentally imposed magnetic fields that may differ substantially from the amplitude and temporal structure of natural disturbances. The analysis also incorporated several complementary safeguards, including fixed primary and sensitivity exposure definitions, leave-one-year-out seasonal adjustment, pairwise matching of seasonal-adjustment methods, spatial cluster bootstrap, three temporal-surrogate models, environmental-window matrices, Shapley/LMG decomposition, Kp and F10.7 comparisons, geographic controls, LAI diagnostics, and land-cover stratification. Importantly, the observation day was excluded from the exposure window by a fixed rule, the temporal alignment was not shifted to maximize the SII–SIF association, and observations were not removed as statistical outliers on the basis of SIF or SII magnitude.

The study includes multiple samples, temperature categories, exposure windows, environmental specifications, land-cover classes, and supplementary outcomes. The primary 28-day, 771 nm harmonic analysis was defined independently of the descriptive 1–90-day scan, which was not used to select an optimal exposure window, lag, or minimum *p*-value. The remaining stratified and supplementary analyses were used to examine sensitivity and heterogeneity rather than to redefine the primary result. Nevertheless, the study remains exploratory, and patterns observed in secondary strata require independent confirmation.

The use of a single direct 771 nm SIF retrieval as the primary outcome also limited multiplicity and provided a consistent reference analysis. Repeated subdivision by wavelength, temperature, vegetation class, geography, and exposure window reduces sample size within individual strata and increases the number of analytical comparisons, making weak associations harder to characterize precisely. This does not make simpler analyses intrinsically more valid, but it does make extensive stratified results more susceptible to sampling variability and overinterpretation. The additional wavelengths and ecological subdivisions were therefore treated as supplementary analyses rather than as independent tests of the primary association.

A key limitation pertains to seasonal adjustment: flexible empirical smoothers may remove not only nonlinear phenological structure but also SII-associated variation that covaries with that structure. In the pairwise-matched sample, the cyclic-spline model substantially reduced the magnitude of the 28-day association relative to harmonic adjustment, although the direction remained negative. This attenuation demonstrates sensitivity to the definition of the seasonal baseline; it does not establish whether the harmonic model retained residual confounding or the spline removed part of an SII-associated component.

Further limitations include dependence on a single satellite mission, irregular OCO-2 sampling, incomplete representation of nonlinear phenology and environmental covariation, the use of global geomagnetic indices rather than local magnetic measurements, possible residual spatial and temporal dependence, and the absence of direct physiological measurements. Temporal-surrogate inference also depended on the structure preserved by the null model, with stronger evidence under circular-shift and block-permutation nulls than under year permutation.

These limitations do not negate the observed weak negative temporal association, but they constrain its interpretation. The present data do not establish causality, identify the relevant local magnetic exposure, or determine a molecular mechanism. Independent observational replication and controlled experiments are therefore needed to establish the generality and biological basis of the phenomenon.

### 4.10. Priorities for Independent Validation

Independent observational replication might use SIF products from other satellite instruments and retrieval algorithms, ideally covering different spatial resolutions, overpass times, and sampling structures. Ground-based SIF, eddy-covariance measurements, conventional chlorophyll-fluorescence parameters, and direct measurements of photosynthetic gas exchange would provide more mechanistically informative outcomes.

The most decisive next step is controlled experimentation using magnetic perturbations that reproduce the scale and temporal structure of natural geomagnetic variability. Experiments should apply changes in the tens-to-hundreds-of-nanotesla range superimposed on the local geomagnetic background, rather than substitute millitesla- or tesla-scale fields. The background field intensity and direction, perturbation amplitude, waveform, frequency content, duration, intermittency, and rate of change should be measured and controlled explicitly. Exposure waveforms should be recorded directly and compared with sham controls using otherwise identical coils and environmental conditions. Experiments using both simplified perturbations and recorded or reconstructed natural geomagnetic-disturbance waveforms would help determine which physical characteristics of the exposure are biologically relevant.

Physiological validation should likewise focus on responses occurring within the range of magnetic conditions that plants encounter naturally. From an evolutionary perspective, any environmentally relevant magnetic sensitivity would operate against the persistent geomagnetic background and its naturally occurring fluctuations; this does not imply that such sensitivity is adaptive, but it provides a strong rationale for testing the natural exposure range before extrapolating from much larger laboratory fields. Dose–response experiments spanning background variability through moderate geomagnetic disturbances would be particularly informative for determining whether a response has a threshold, scales continuously with perturbation amplitude, or is restricted to a particular temporal or spectral regime.

Experiments should also test whether responses depend on physiological context. Relevant factors include low versus optimal temperature, light intensity, acclimation state, antioxidant capacity, and the redox state of the photosynthetic electron-transport chain. Measurements should include chlorophyll-fluorescence parameters, gas exchange or oxygen evolution, P700 redox kinetics, reactive oxygen species, antioxidant responses, and growth or viability endpoints. Cryptochrome mutants, electron-transport inhibitors, alternative electron acceptors, and redox-active treatments could then be used to distinguish among mechanistic hypotheses.

A biological interpretation of the observed SII–SIF association would be strengthened if controlled exposures within the natural geomagnetic range produced a small, directionally consistent physiological response under the environmental conditions suggested by the observational analysis. Reproducibility across independent laboratories would be particularly informative. This would provide evidence that the response depends systematically on physically defined exposure parameters rather than simply on the presence or absence of an imposed magnetic treatment. Failed reproducibility under well-controlled, environmentally realistic conditions would instead weaken a biological interpretation and increase concern about residual confounding or satellite-related explanations.

## 5. Conclusions

This global observational analysis identified a weak negative temporal association between a multi-week Dst-derived geomagnetic-disturbance index and seasonally adjusted OCO-2 SIF at 771 nm. Under the primary harmonic specification, the association was observed in the pooled dataset and was more negative in several vegetated and lower-to-intermediate-temperature strata, whereas estimates for the Barren land-cover class and the Sahara geographic control were close to zero or substantially weaker. The SII coefficient also remained predominantly negative across a broad range of simultaneous PAR and VPD adjustment specifications. Nevertheless, the magnitude of the association was small relative to the variability of individual SIF observations, and the contribution assigned to SII accounted for less than one percentage point of explained variance across all temperature regimes examined.

The strength and statistical significance of this relationship varied considerably depending on the analytical assumptions made during the study. Flexible cyclic-spline seasonal adjustment substantially attenuated the estimate while preserving its negative direction, and temporal-surrogate support differed among null models. The temporal-surrogate analyses provided mixed evidence: the observed association was inconsistent with the circular-shift and 30-day block-permutation null models but remained compatible with the year-permutation null. Consequently, the consistently negative direction of the relationship was more robust than its specific magnitude or statistical significance, indicating sensitivity to the treatment of seasonal and other temporally structured variation.

This study identified a context-dependent and method-sensitive association between geomagnetic indices and SIF anomalies. This association is modulated by ecological context and analytical approach. Future work should focus on verifying these findings through independent cross-sensor measurements using distinctly processed SIF data. Independent verification should also incorporate local geomagnetic measurements and controlled experiments that mimic natural magnetic field fluctuations in terms of strength, shape and timing.

## Figures and Tables

**Table 1:** Analytical samples and control definitions.

Sample/Control	Operational Definition	Analytical Role and Interpretation
Full sample	All land retrievals at absolute latitudes below 60°, aggregated to daily 0.5° cell means, that satisfied the SIF and ancillary-data quality criteria, exact MODIS matching, detrending, exposure-availability, and complete-case requirements for the corresponding analysis.	Primary spatially extensive land sample within the predefined latitude domain.
Persistently vegetated sample	Cells with median LAI ≥ 1.0 and 10th-percentile LAI ≥ 0.30.	Functional vegetation sample intended to retain cells with consistently present vegetation rather than episodic or strongly seasonal greenness.
Strict low-LAI control	Cells with median LAI ≤ 0.10 and 90th-percentile LAI ≤ 0.30.	Restrictive functional low-vegetation control containing very few cells; used descriptively.
Barren land-cover class	Cells assigned to the dominant 2022 MCD12C1 IGBP Barren class [55].	Principal land-cover-based barren comparison.
Sahara geographic control	Rectangular region bounded by 15–32° N and 18° W–40° E.	Geographic low-vegetation diagnostic.
South Atlantic Anomaly diagnostic	Rectangular geographic approximation bounded by 50° S–0° N and 90° W–10° E.	Supplementary diagnostic of possible particle-related satellite artefacts [57,58]. The box was neither a precise physical boundary of the South Atlantic Anomaly nor a measure of local magnetic exposure, and it could not exclude other instrumental, orbital, atmospheric, or sampling artefacts.

**Table 2:** Primary and sensitivity estimates of the association between trailing-mean SII exposure and residual OCO-2 SIF at 771 nm.

Analytical Sample	Seasonal Adjustment	SII Window (Days)	Spearman ρ	95% Cluster-Bootstrap CI	OLS Change per 100 nT	Observations	Spatial Cells	Maximum Empirical *p*
Full sample	Harmonic	28	−0.051	[−0.053, −0.050]	−0.0381	1,949,342	24,304	0.084
Full sample, pairwise matched	Cyclic spline	28	−0.013	[−0.014, −0.012]	−0.0091	1,947,618	24,299	0.127
Full sample	Harmonic	21	−0.045	[−0.047, −0.044]	−0.0315	1,949,342	24,304	0.128
Persistently vegetated sample	Harmonic	28	−0.028	[−0.030, −0.025]	−0.0187	493,361	6736	0.036
Sahara geographic control	Harmonic	28	0.012	[0.006, 0.018]	0.0019	93,893	854	0.842

Notes: OLS slope estimates are expressed in residual-SIF units per 100 nT increase in trailing-mean SII. Confidence intervals were obtained by resampling 0.5° spatial cells with replacement while retaining all observations from each selected cell. The maximum empirical *p*-value is the largest of the two-sided plus-one *p*-values from year permutation, leap-year-aware circular shift, and 30-day block permutation. It is reported only as a conservative summary of the three temporal null models and is not a combined *p*-value.

## Data Availability

The OCO-2 Level 2 Lite SIF data (V11r/V11.2r) are available from NASA GES DISC (https://disc.gsfc.nasa.gov; accessed on 18 February 2026). The OMNI2 geomagnetic indices and F10.7 cm solar radio flux are available from NASA/GSFC’s Space Physics Data Facility (https://omniweb.gsfc.nasa.gov; accessed on 22 November 2025). ERA5 reanalysis data are available from the Copernicus Climate Change Service (https://cds.climate.copernicus.eu; accessed on 18 February 2026). The MODIS/Terra MOD15A2H Collection 6.1 LAI/FPAR product (accessed on 18 February 2026) and the 2022 MCD12C1 Collection 6.1 Land Cover Type product (IGBP classification; accessed on 25 July 2026) are available from NASA LP DAAC (https://lpdaac.usgs.gov). All analysis code, validated analytical outputs, and figure-generation scripts are available at Zenodo (DOI: 10.5281/zenodo.21768818).

## Supplementary Materials

The supporting information can be downloaded at https://www.mdpi.com/article/10.3390/biology15161415/s1: Table S1. Data sources, temporal coverage, spatial harmonization, and principal eligibility rules; Table S2. Operational definitions and sizes of principal analytical samples and controls; Table S3. Temperature-stratified SII–SIF estimates in the full harmonic 28-day sample; Table S4. Temporal-surrogate inference for the primary full-sample harmonic 28-day analysis; Table S5. Selected geomagnetic, solar, geographic, and low-vegetation sensitivity comparisons; Table S6. Design and interpretation of the environmental-driver specification matrix; Table S7. Temperature-stratified Shapley/LMG decomposition at the common 28-day trailing window; Table S8. Pooled harmonic 28-day estimates by dominant MCD12C1 land-cover class; Table S9. LAI-based analytical definitions and inferential role; Table S10. Selected fixed-window associations for supplementary SIF outcomes; Table S11. Analytical hierarchy and interpretation of the reported analyses; Figure S1. Spatial coverage and analytical sample definitions on the 0.5° × 0.5° analysis grid; Figure S2. Descriptive dependence of the SII–SIF association on trailing-window length; Figure S3. Detailed temporal-surrogate distributions for the primary 28-day harmonic analysis; Figure S4. Biological, geographic, and low-vegetation sensitivity controls; Figure S5. Matched-sample comparison of associations obtained with SII, Kp, and F10.7; Figure S6. Environmental-driver coefficient matrix across SII, PAR, and VPD trailing-window specifications; Figure S7. Complete temperature-stratified Shapley/LMG decomposition at the common 28-day trailing window; Figure S8. LAI and temperature composition of the land-cover groups and exploratory LAI-based heterogeneity; Supplementary Data S1. Fixed-window estimates; Supplementary Data S2. Temporal-surrogate inference; Supplementary Data S3. Exposure-window scan; Supplementary Data S4. Environmental-driver adjustment; Supplementary Data S5. Shapley/LMG decomposition; Supplementary Data S6. Ecological stratification and sample composition.
