# Supplementary material for: A Weak Temporal Association Between Multi-Week Geomagnetic Activity and Satellite-Derived Solar-Induced Chlorophyll Fluorescence Anomalies
Source: Biology (Basel). 2026 Aug 18;15(16):1415. doi: 10.3390/biology15161415 (PMC13509825; doi:10.3390/biology15161415)
Supplement: Supplementary file 1 [file biology-15-01415-s001.zip › supplementary_mdpi_v2.3_complete.pdf]

Supplementary Materials

This document contains the supplementary tables, figures, and machine-readable data files supporting the analyses reported in the manuscript. The numerical summaries below reproduce validated outputs; larger result grids and realization-level outputs are provided as Supplementary Data files.

Supplementary Tables

Table S1. Data sources, temporal coverage, spatial harmonization, and principal eligibility rules.

Data source	Product or index	Native temporal support	Role in the analysis	Principal processing or eligibility rule
OCO-2	Level 2 Lite SIF V11r/V11.2r	Sounding level; September 2014–December 2024	Primary SIF at 771 nm; supplementary SIF at 757 nm and provider-derived 740 nm	Land retrievals; cloud fraction ≤60%; aerosol fraction ≤50%; LAI ≥0.01; |latitude|<60°; accepted soundings aggregated to daily 0.5° cell means
NASA OMNI	Dst	Hourly	Source of daily SII	Daily mean Dst sign-inverted; observation day excluded before calculation of trailing means
NASA OMNI	Kp	3-hourly	Related geomagnetic-index sensitivity analysis	Stored 0.1-Kp units multiplied by 0.1; daily series processed using the same shift-before-rolling convention
NASA OMNI	F10.7-cm solar radio flux	Daily	Solar-activity comparator	Processed using the same shift-before-rolling convention; matched samples used for direct comparisons
ERA5	2-m air temperature and 2-m dew-point temperature	Hourly	Temperature stratification and VPD	Aggregated to daily values; VPD calculated using the Magnus formula and expressed in kPa
ERA5	Surface solar radiation downwards	Hourly	PAR energy proxy	Calculated as 0.45×SSRD/3600 for each hourly record

Data source	Product or index	Native temporal support	Role in the analysis	Principal processing or eligibility rule
				and then averaged daily; units W m−2
MODIS/Terra	MOD15A2H Collection 6.1 LAI	Eight-day composite	Eligibility, exact matching, persistent-vegetation and LAI analyses	Equal-overlap remapping from the offset 0.5° ICDC grid; central-only exact cell/composite matching for the primary input
MODIS/Terra	MCD12C1 Collection 6.1, 2022 IGBP class	Annual	Dominant land-cover classification	Dominant class assigned to each 0.5° analysis-grid cell

Table S2. Operational definitions and sizes of principal analytical samples and controls.

Sample or control	Operational definition or specification	Observations	Spatial cells	Analytical role
Full sample, harmonic, 28 days	Eligible land observations with valid 771-nm harmonic residuals and 28-day SII exposure	1,949,342	24,304	Primary analysis
Full sample, pairwise matched, cyclic spline, 28 days	Observations with valid harmonic and cyclic-spline residuals	1,947,618	24,299	Direct seasonal-adjustment sensitivity comparison
Persistently vegetated	Median LAI ≥1.0 and 10th-percentile LAI ≥0.30	493,361	6,736	Functional vegetation comparison
Strict low-LAI control	Median LAI ≤0.10 and 90th-percentile LAI ≤0.30	2,470	26	Descriptive functional low-vegetation control; not the Barren land-cover class
MCD12C1 Barren class	Dominant 2022 IGBP Barren class	71,511	731	Systematic land-cover comparison
Sahara geographic control	15°–32°N, 18°W–40°E	93,893	854	Geographic low-vegetation diagnostic
South Atlantic Anomaly diagnostic	50°S–0°N, 90°W–10°E	220,050	2,778	Geographic diagnostic of possible particle-related satellite artifacts

Note: Observation and cell counts refer to the pooled harmonic 28-day analysis unless another specification is stated. Fixed-window estimates for the strict low-LAI subset are provided in Supplementary Data S1. Its small number of spatial cells limits stable conventional inference.

Table S3. Temperature-stratified SII–SIF estimates in the full harmonic 28-day sample.

Temperature regime	Operational range	Spearman ϱ	95% spatial cluster-bootstrap CI	Observations	Spatial cells
Frozen	T<−1°C	−0.106	[−0.109, −0.101]	160,994	9,957
Cold	−1°C≤T≤7°C	−0.051	[−0.055, −0.047]	186,306	13,151
Cool	7°C<T≤15°C	−0.076	[−0.079, −0.073]	374,155	17,794
Optimum	15°C<T≤25°C	−0.055	[−0.058, −0.053]	712,536	22,033
Warm stress	25°C<T≤30°C	−0.008	[−0.010, −0.005]	366,069	14,834
Extreme heat	T>30°C	0.005	[−0.000, 0.009]	149,282	7,170

Note: Temperature was the trailing 10-day mean of ERA5 2-m air temperature. The categories are operational ecological strata rather than universal physiological thresholds. Complete temporal-surrogate outputs for these strata are provided in Supplementary Data S2.

Table S4. Temporal-surrogate inference for the primary full-sample harmonic 28-day analysis.

Null model	Temporal structure retained	Realizations	Observed ϱ	Two-sided plus-one empirical p
Year permutation	Within-year daily organization; leap and non-leap years permuted separately	1,000	−0.051	0.084
Leap-year-aware circular shift	Complete exposure sequence and autocorrelation	1,000	−0.051	0.011
30-day block permutation	Short-range organization within 30-day blocks	1,000	−0.051	0.001
Maximum empirical p	Conservative summary of the three separate null models	—	−0.051	0.084

Note: The maximum empirical p-value is not a combined probability or an additional statistical test. The primary association was more extreme than the circular-shift and block-permutation distributions but remained compatible with the year-permutation null.

Table S5. Selected geomagnetic, solar, geographic, and low-vegetation sensitivity comparisons.

Comparison	Exposure or region	Seasonal adjustment	Window	Spearman ϱ	Observations	Spatial cells	Interpretation
Primary SII	Dst-derived SII	Harmonic	28 days	−0.051	1,949,342	24,304	Primary specification

Comparison	Exposure or region	Seasonal adjustment	Window	Spearman ϱ	Observations	Spatial cells	Interpretation
Kp	Planetary geomagnetic index	Harmonic	28 days	−0.033	1,949,342	24,304	Related geomagnetic proxy, not independent replication
F10.7	Solar radio flux	Harmonic	28 days	−0.012	1,921,063	24,300	Comparator for broader solar variability
Sahara	Geographic box	Harmonic	28 days	0.012	93,893	854	Geographic low-vegetation diagnostic
South Atlantic Anomaly	Geographic box	Harmonic	28 days	−0.014	220,050	2,778	Diagnostic of possible particle-related satellite artifacts
MCD12C1 Barren	Dominant land-cover class	Harmonic	28 days	0.010	71,511	731	Systematic land-cover-based barren comparison

Note: These comparisons have different physical units and purposes. Their regression slopes are therefore not compared in this summary. Conventional sample-level p-values are omitted because they are not the principal inferential framework.

Table S6. Design and interpretation of the environmental-driver specification matrix.

Component	Specification	Number of alternatives	Summary retained
SII trailing window	1–28 days	28	Median adjusted SII coefficient, interquartile range, sign stability, and valid-model count
PAR trailing window	1–28 days for each SII window	28	Evaluated jointly with each VPD window
VPD trailing window	1–28 days for each SII window	28	Evaluated jointly with each PAR window
PAR–VPD combinations per SII window	28×28	784	Environmental-window sensitivity at a fixed SII window

Component	Specification	Number of alternatives	Summary retained
Total fitted models per analytical stratum	28×28×28	21,952	Full coefficient grid supplied in Supplementary Data S4

Note: The matrix was designed to test whether the estimated SII coefficient depended on one selected PAR or VPD accumulation window. It was not used to choose a best environmental window and does not eliminate residual environmental confounding.

Table S7. Temperature-stratified Shapley/LMG decomposition at the common 28-day trailing window.

Temperature regime	SII contribution	PAR contribution	VPD contribution	Full-model R2
Frozen	0.4423	6.6512	2.2553	9.3488
Cold	0.1363	0.9697	0.0240	1.1301
Cool	0.2476	0.1580	0.0118	0.4174
Optimum	0.1008	0.0279	0.4390	0.5678
Warm stress	0.0098	0.2628	0.1816	0.4542
Extreme heat	0.0159	0.1319	0.0120	0.1598

Note: Values are absolute R^2 percentage points and sum to the full-model R^2, apart from rounding. The decomposition is model-specific and descriptive, not causal.

Table S8. Pooled harmonic 28-day estimates by dominant MCD12C1 land-cover class.

Land-cover class	Spearman ϱ	Descriptive parametric p	Observations	Spatial cells
Forest	−0.121	7.33×10−168	51,888	850
Cropland	−0.078	9.60×10−62	45,276	640
Savanna	−0.055	1.90×10−26	37,606	513
Shrubland/Savanna	−0.048	1.56×10−44	86,319	994
Grassland	−0.044	1.21×10−59	135,679	1,598
Barren	0.010	0.007	71,511	731

Note: The parametric p-values are descriptive diagnostics and are not the principal inference because observations are spatially and temporally dependent. Fixed-window land-cover estimates and their temporal-surrogate summaries are supplied in Supplementary Data S6. Barren denotes only the actual MCD12C1 land-cover class and not the strict low-LAI control.

Table S9. LAI-based analytical definitions and inferential role.

LAI analysis	Definition	Inferential role
Persistently vegetated	Median LAI ≥1.0 and 10th-percentile LAI ≥0.30	Predefined functional vegetation sample
Strict low-LAI control	Median LAI ≤0.10 and 90th-percentile LAI ≤0.30	Descriptive low-vegetation control with very few cells
Median-LAI quartiles Q1–Q4	Quartiles of the cell-level median-LAI distribution, using unrounded analytical cut points	Exploratory heterogeneity analysis; not a dose–response test

Note: The final quartile estimates, cluster-bootstrap confidence intervals, sample sizes, and land-cover/LAI composition outputs are provided in Figure S8 and Supplementary Data S6. The

unrounded quartile cut points were not retained as a separate machine-readable output in the validated analysis run.

Table S10. Selected fixed-window associations for supplementary SIF outcomes.

SIF outcome	Seasonal adjustment	SII window	Spearman ϱ	Observations
771 nm	Harmonic	28 days	−0.051	1,949,342
771 nm	Harmonic	21 days	−0.045	1,949,342
771 nm	Cyclic spline, pairwise matched	28 days	−0.013	1,947,618
Provider-derived 740 nm	Harmonic	28 days	−0.047	1,949,342
Provider-derived 740 nm	Harmonic	21 days	−0.041	1,949,342
Provider-derived 740 nm	Cyclic spline	28 days	−0.004	1,947,618
Provider-derived 740 nm	Cyclic spline	21 days	−0.003	1,947,618
757 nm	Harmonic	28 days	−0.034	1,949,342
757 nm	Harmonic	21 days	−0.028	1,949,342
757 nm	Cyclic spline	28 days	0.009	1,947,618
757 nm	Cyclic spline	21 days	0.009	1,947,618

Note: The 740-nm variable was provider-derived from the direct SIF retrievals and does not constitute independent wavelength or sensor replication. The 757- and 771-nm retrievals were obtained from the same instrument and share atmospheric, temporal, spatial, and sampling structure.

Table S11. Analytical hierarchy and interpretation of the reported analyses.

Analytical component	Outcome or sample	Exposure and model	Role
Primary specification	OCO-2 SIF at 771 nm, full sample	28-day SII trailing mean; leave-one-year-out harmonic adjustment	Primary estimate
Shorter-window sensitivity	OCO-2 SIF at 771 nm, full sample	21-day SII trailing mean; harmonic adjustment	Predefined sensitivity analysis
Seasonal-adjustment sensitivity	Pairwise-matched full sample	28-day SII; leave-one-year-out cyclic spline	Sensitivity to seasonal baseline
Spatial uncertainty	Principal fixed-window estimates	1,000 cell-cluster bootstrap realizations	Graphical and tabular confidence intervals
Temporal inference	Principal and supplementary strata	Year permutation, circular shift, and 30-day block permutation	Empirical temporal null models
Window-length analysis	OCO-2 SIF at 771 nm	Descriptive 1–90-day scan	Descriptive only; no optimal-window selection
Environmental adjustment	SIF residuals	SII, PAR, and VPD window matrix	Exploratory specification sensitivity

Analytical component	Outcome or sample	Exposure and model	Role
Variance decomposition	SIF residuals	Common 28-day SII, PAR, and VPD model	Descriptive Shapley/LMG attribution within the specified model
Ecological heterogeneity	Temperature, LAI, and land-cover strata	Primarily 28-day harmonic estimates	Exploratory heterogeneity
Geographic and low-vegetation diagnostics	Sahara, SAA, strict low-LAI, and Barren comparisons	Primarily 28-day harmonic estimates	Diagnostic or descriptive controls
Alternative indices	Kp and F10.7	28-day trailing means on matched samples	Sensitivity comparisons, not independent replications
Supplementary SIF outcomes	Direct 757 nm and provider-derived 740 nm	21- and 28-day harmonic and spline estimates	Internal spectral consistency analyses

Supplementary Figures

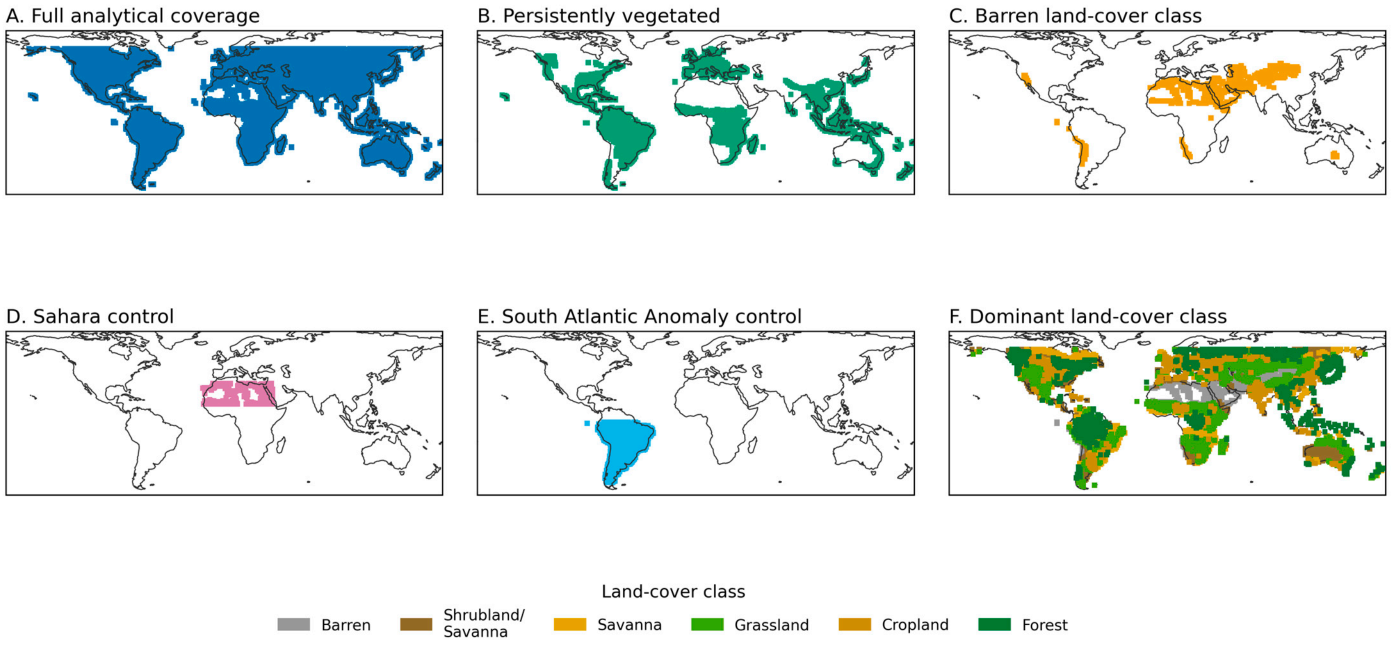

Figure S1

Figure S1. Spatial coverage and analytical sample definitions on the 0.5° × 0.5° analysis grid. Maps show the spatial cells contributing to the full primary sample and the principal vegetation and geographic comparisons, including persistently vegetated cells, the MCD12C1 Barren land-cover class, the Sahara geographic control (15–32° N, 18° W–40° E), and the rectangular South Atlantic Anomaly diagnostic (50° S–0°, 90° W–10° E). The strict low-LAI control is a separate functional definition based on the LAI distribution and is not equivalent to the Barren class. Geographic boxes are descriptive diagnostics rather than local magnetic-exposure measurements.

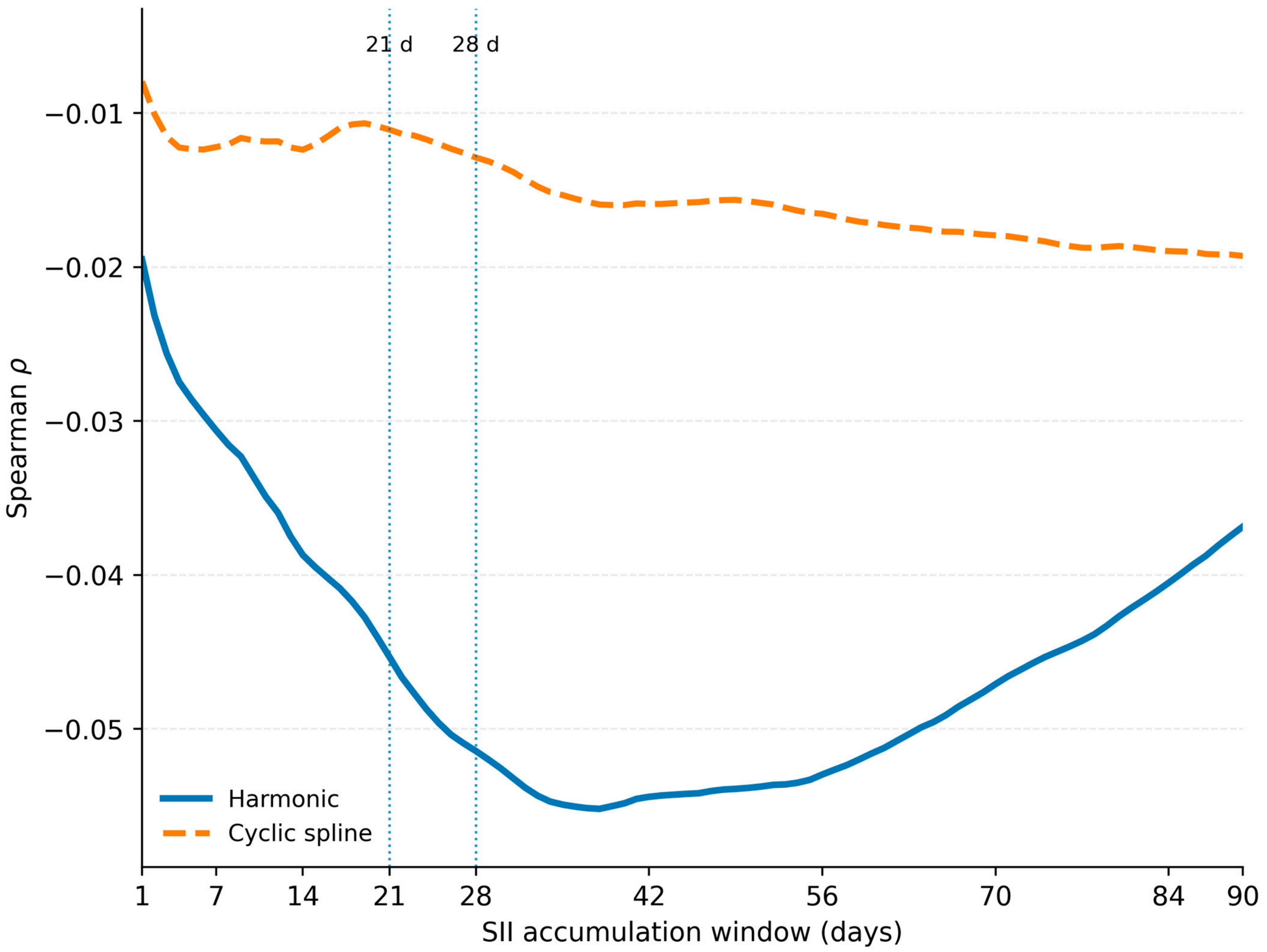

Figure S2

Figure S2. Descriptive dependence of the SII–SIF association on trailing-window length. Spearman correlations and effect-size summaries are shown for SII trailing means from 1 to 90 days, with the SIF-observation day excluded before rolling. Curves compare leave-one-year-out harmonic residuals, cyclic B-spline residuals, and the pairwise-matched observation set, as indicated. The 28-day primary and 21-day sensitivity windows were specified independently of this scan. The figure is descriptive and was not used to select an optimal window, a minimum p-value, or a biological integration timescale.

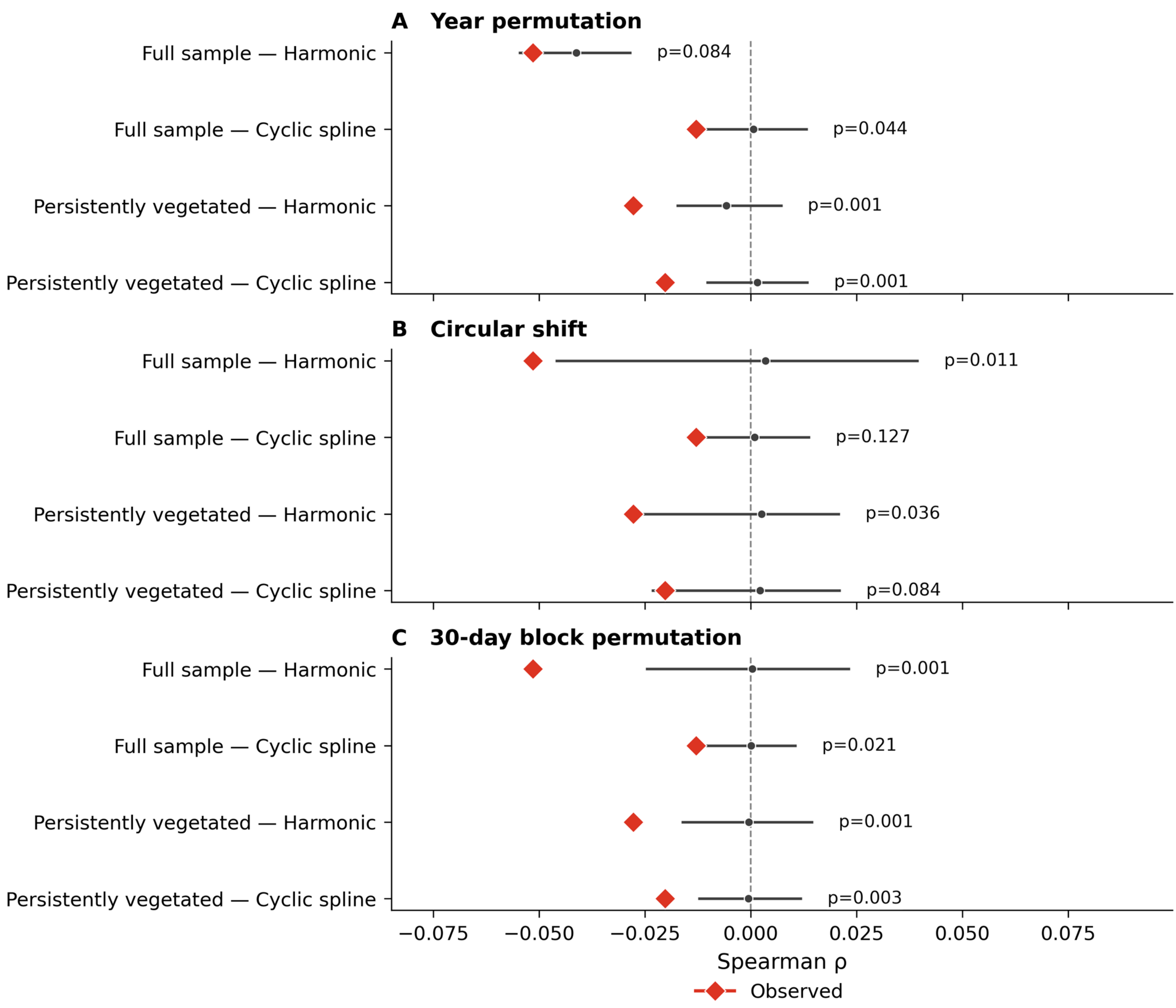

Figure S3

Figure S3. Detailed temporal-surrogate distributions for the primary 28-day harmonic analysis. The observed Spearman coefficient for OCO-2 SIF at 771 nm is compared with distributions from 1,000 year permutations, 1,000 leap-year-aware circular shifts, and 1,000 30-day block permutations of the daily SII series, followed in each case by the same shift-before-rolling exposure construction. Vertical reference lines indicate the observed coefficient. Two-sided plus-one empirical p-values were 0.084, 0.011, and 0.001, respectively. The null models preserve different temporal properties and were not combined into a single formal probability.

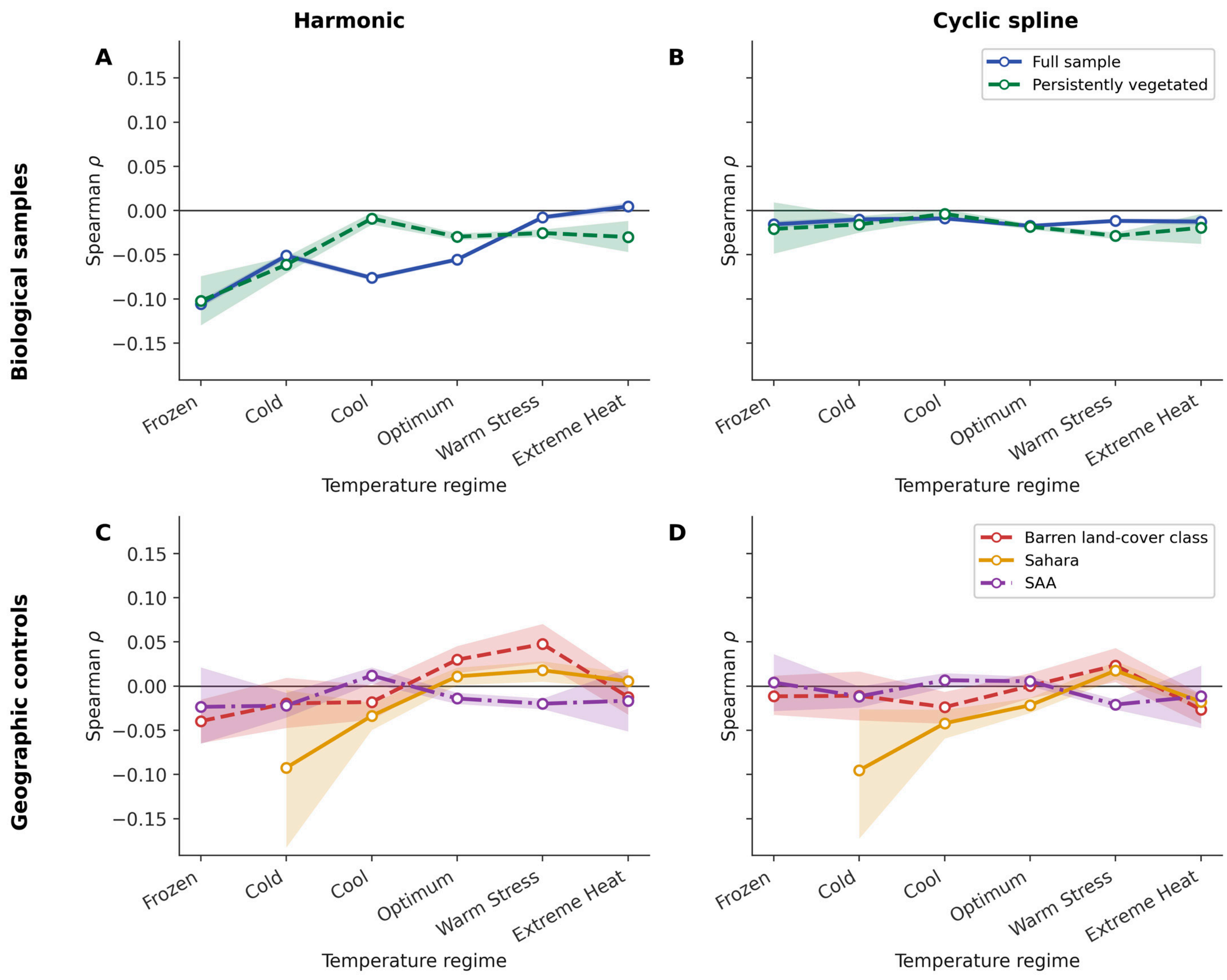

Figure S4

Figure S4. Biological, geographic, and low-vegetation sensitivity controls. Pooled and temperature-stratified SII–SIF estimates are compared among the full sample, persistently vegetated cells, the Sahara geographic control, the strict low-LAI control, and the South Atlantic Anomaly diagnostic, as available. Estimates use the 28-day trailing mean of SII and leave-one-year-out harmonic residuals unless otherwise indicated. The strict low-LAI control contains few spatial cells and is presented descriptively; it is not the MCD12C1 Barren land-cover class. None of the geographic comparisons is a definitive instrumental or biological negative control.

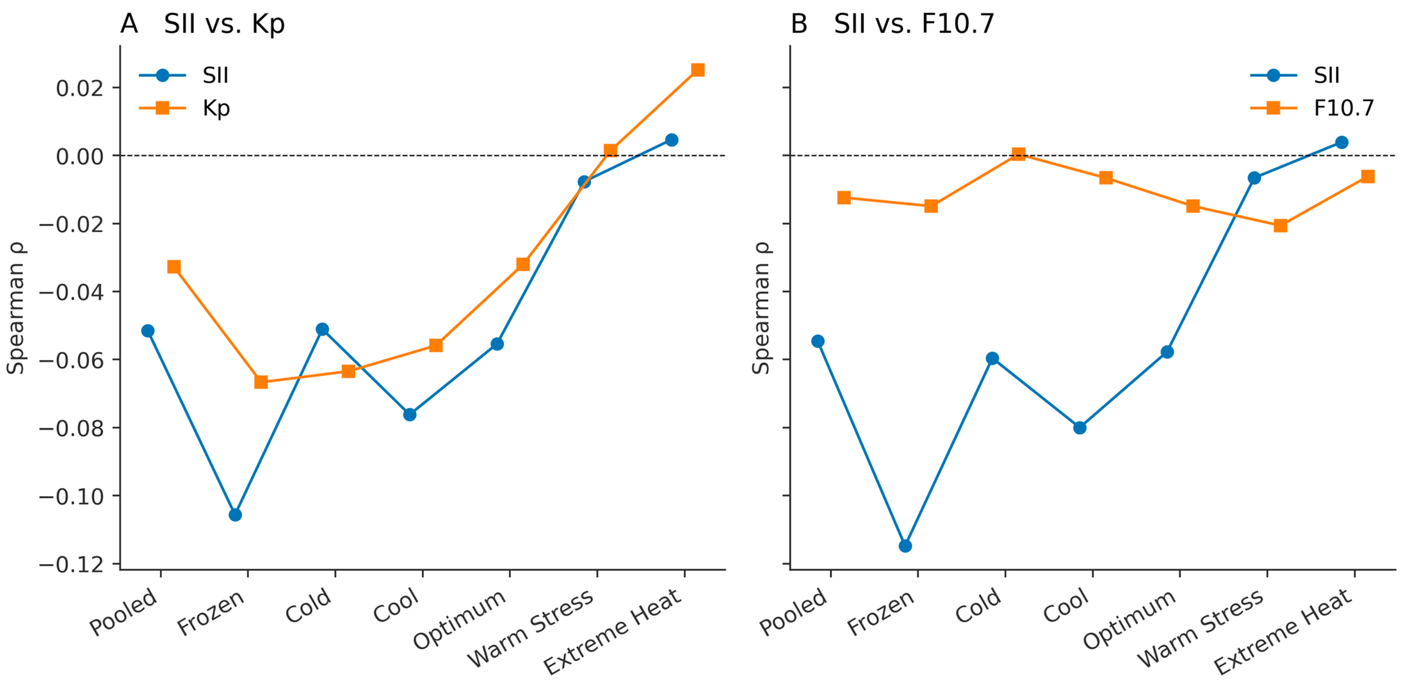

Figure S5

Figure S5. Matched-sample comparison of associations obtained with SII, Kp, and F10.7. Associations with residual OCO-2 SIF at 771 nm are shown for the 28-day trailing means of SII, Kp, and F10.7 on the corresponding matched observation sets. Where exposure availability differed, the SII reference estimate was recalculated using the same observations. Kp and SII are related planetary geomagnetic indices, whereas F10.7 is a solar-activity comparator; these comparisons are sensitivity analyses and do not constitute independent exposure replications.

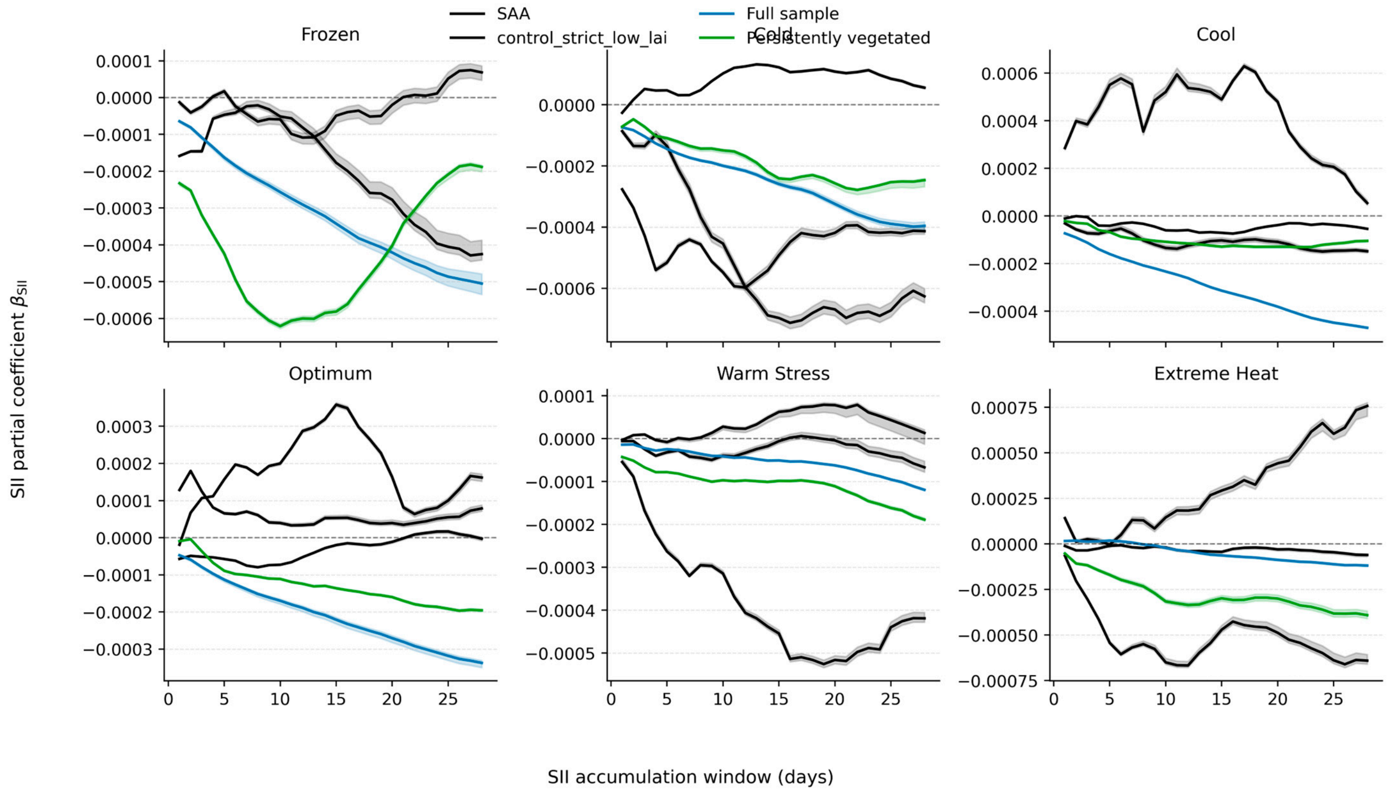

Figure S6

Figure S6. Environmental-driver coefficient matrix across SII, PAR, and VPD trailing-window specifications. The multivariable model included residual SIF as the outcome and SII, the ERA5 SSRD-derived PAR energy proxy, and VPD as simultaneous predictors. For each SII window from 1 to 28 days, PAR and VPD windows independently ranged from 1 to 28 days, producing 784 environmental specifications per SII window and analytical stratum. The figure summarizes the median adjusted SII coefficient, its interquartile range or sign stability, and the number of valid models, as indicated. The matrix assesses specification sensitivity and does not identify a best window or remove all potential environmental confounding.

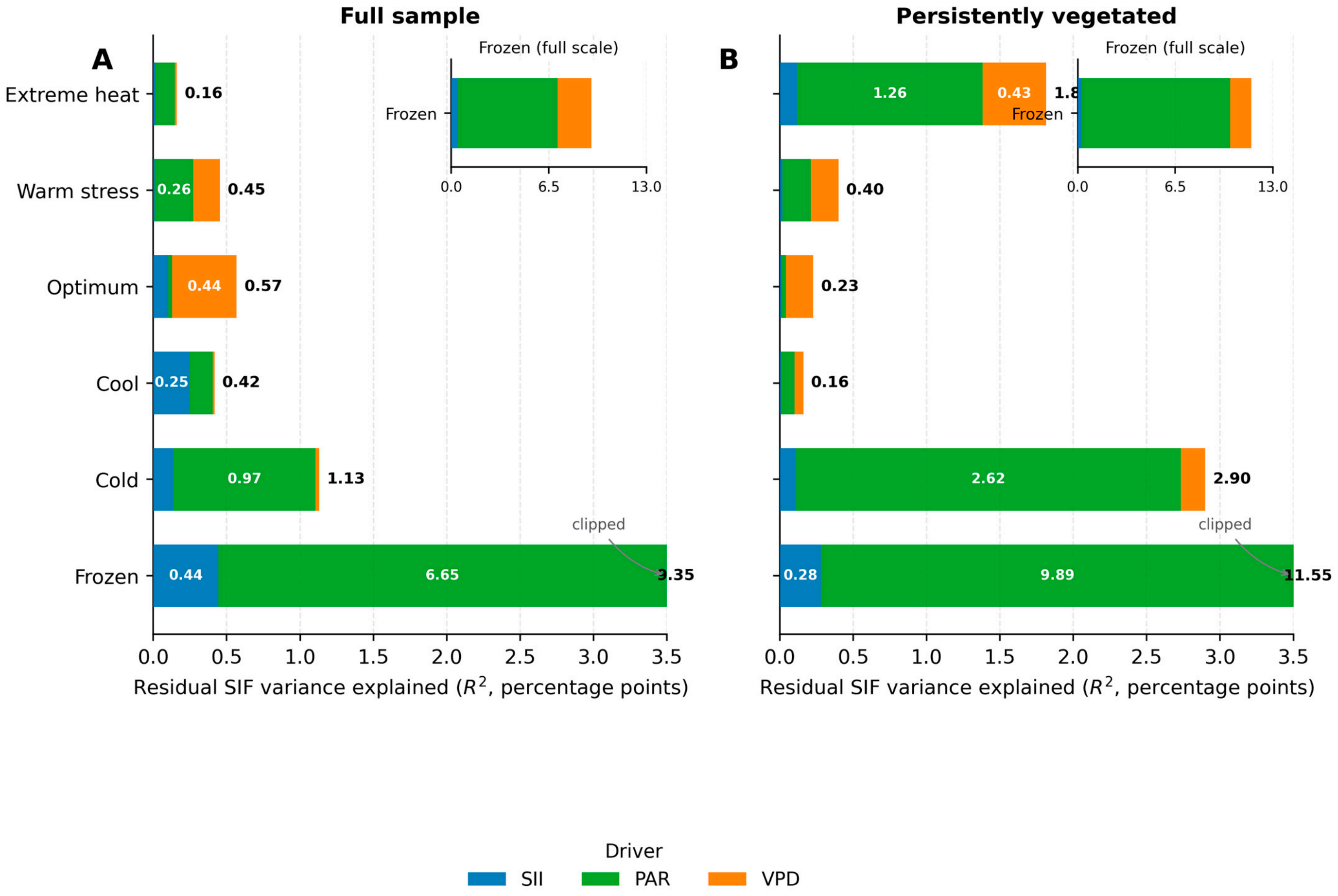

Figure S7

Figure S7. Complete temperature-stratified Shapley/LMG decomposition at the common 28-day trailing window. Absolute contributions of SII, PAR, and VPD to ordinary model R^2 were calculated by exact averaging over all six possible predictor-entry orders. Contributions are expressed as R^2 percentage points and sum to the full-model R^2, apart from rounding. The decomposition is model-specific and explanatory rather than causal; a large relative share within a low-R^2 model does not imply a large absolute association.

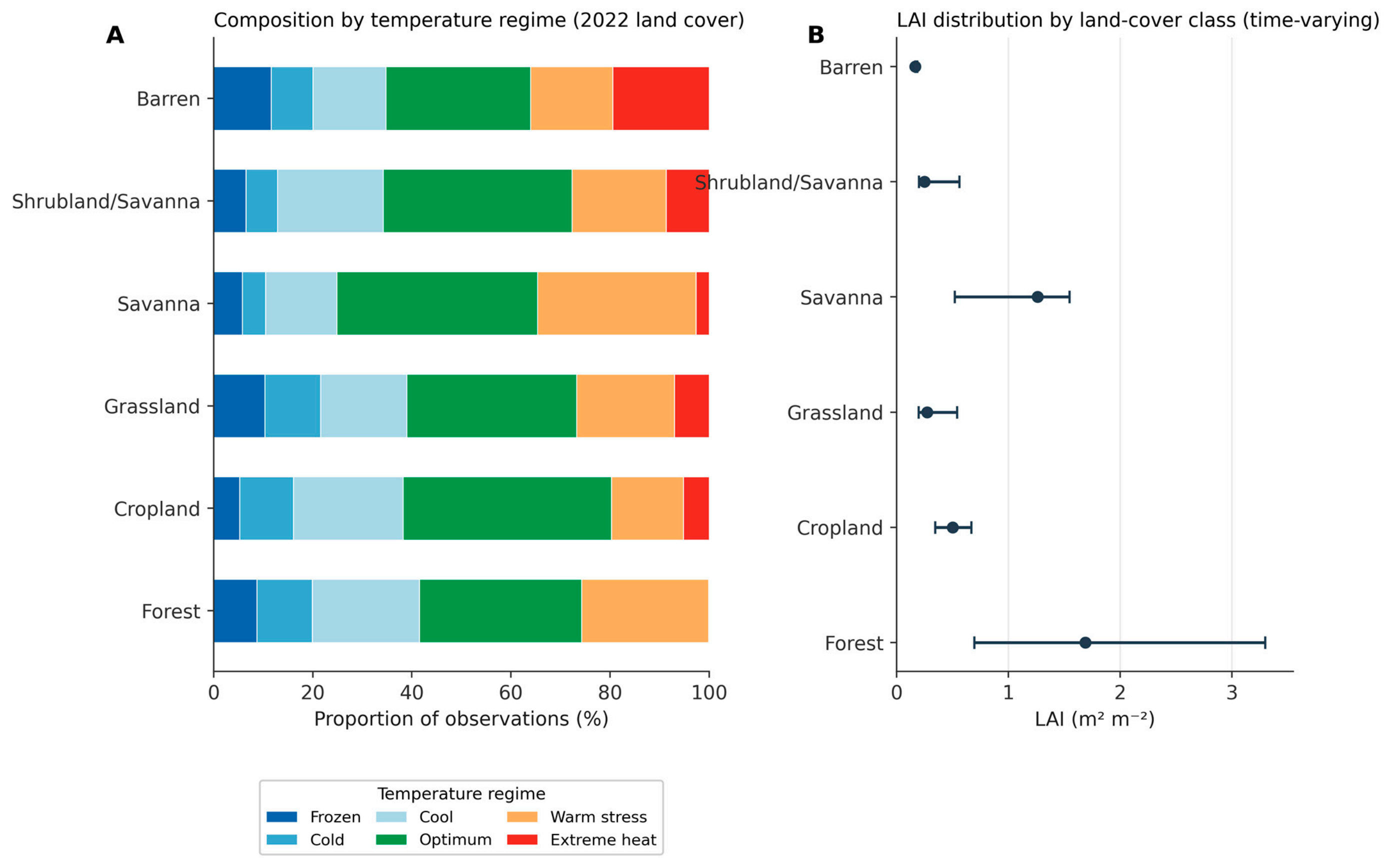

Figure S8

Figure S8. LAI and temperature composition of the land-cover groups and exploratory LAI-based heterogeneity. Panels summarize the distribution of MODIS/Terra MOD15A2H Collection 6.1 LAI and ERA5 temperature regimes within the dominant MCD12C1 land-cover classes and present the associated exploratory LAI-stratified SII–SIF estimates, as indicated. The figure demonstrates that land-cover classes are not environmentally exchangeable and that vegetation-density strata do not constitute a simple monotonic dose–response series. The strict low-LAI control remains distinct from the MCD12C1 Barren class.

Supplementary Data File

All machine-readable supplementary data are provided in a single archive, Supplementary_data.zip. The archive is organized into six directories corresponding to Supplementary Data S1–S6 and includes a file manifest, SHA-256 checksums, and data dictionaries.

- **Supplementary Data S1 — Fixed-window estimates:** fixed-window association estimates, effect-size summaries, and spatial cluster-bootstrap confidence-interval summaries.
- **Supplementary Data S2 — Temporal-surrogate inference:** temporal-surrogate summaries and realization-level null distributions for year permutation, circular shift, and 30-day block permutation.
- **Supplementary Data S3 — Exposure-window scan:** descriptive 1–90-day trailing-window scan for OCO-2 SIF at 771 nm under harmonic, cyclic-spline, and pairwise-matched specifications.
- **Supplementary Data S4 — Environmental-driver adjustment:** PAR–VPD environmental-adjustment coefficient grid and coefficient-stability summaries.
- **Supplementary Data S5 — Shapley/LMG decomposition:** exact Shapley/LMG decompositions for SII, PAR, and VPD.

- **Supplementary Data S6 — Ecological stratification and sample composition:** land-cover fixed-window estimates, land-cover temporal-surrogate summaries and realization-level null distributions, LAI-stratification summaries, analytical-sample composition, and land-cover, LAI, and temperature-composition outputs.
